# Supplementary material for: Structural basis of substrate recognition and allosteric activation of the proapoptotic mitochondrial HtrA2 protease
Source: Nat Commun. 2024 May 30;15:4592. doi: 10.1038/s41467-024-48997-5 (PMC11535027; doi:10.1038/s41467-024-48997-5)
Supplement: Supplementary file 1 — Supplementary Information [file 41467_2024_48997_MOESM1_ESM.pdf]

**Supplementary information**

**for**

# Structural basis of substrate recognition and allosteric activation of the proapoptotic mitochondrial HtrA2 protease

Emelie E. Aspholm[1,2], Jens Lidman[1,2], Björn M. Burmann[1,2]

[1] Department of Chemistry and Molecular Biology, University of Gothenburg, 405 30 Göteborg, Sweden

[2] Wallenberg Centre for Molecular and Translational Medicine, University of Gothenburg, 405 30 Göteborg, Sweden

*Correspondence should be addressed to BMB:

Tel: +46-317863937; e-mail: bjorn.marcus.burmann@gu.se

## Supplementary Figures

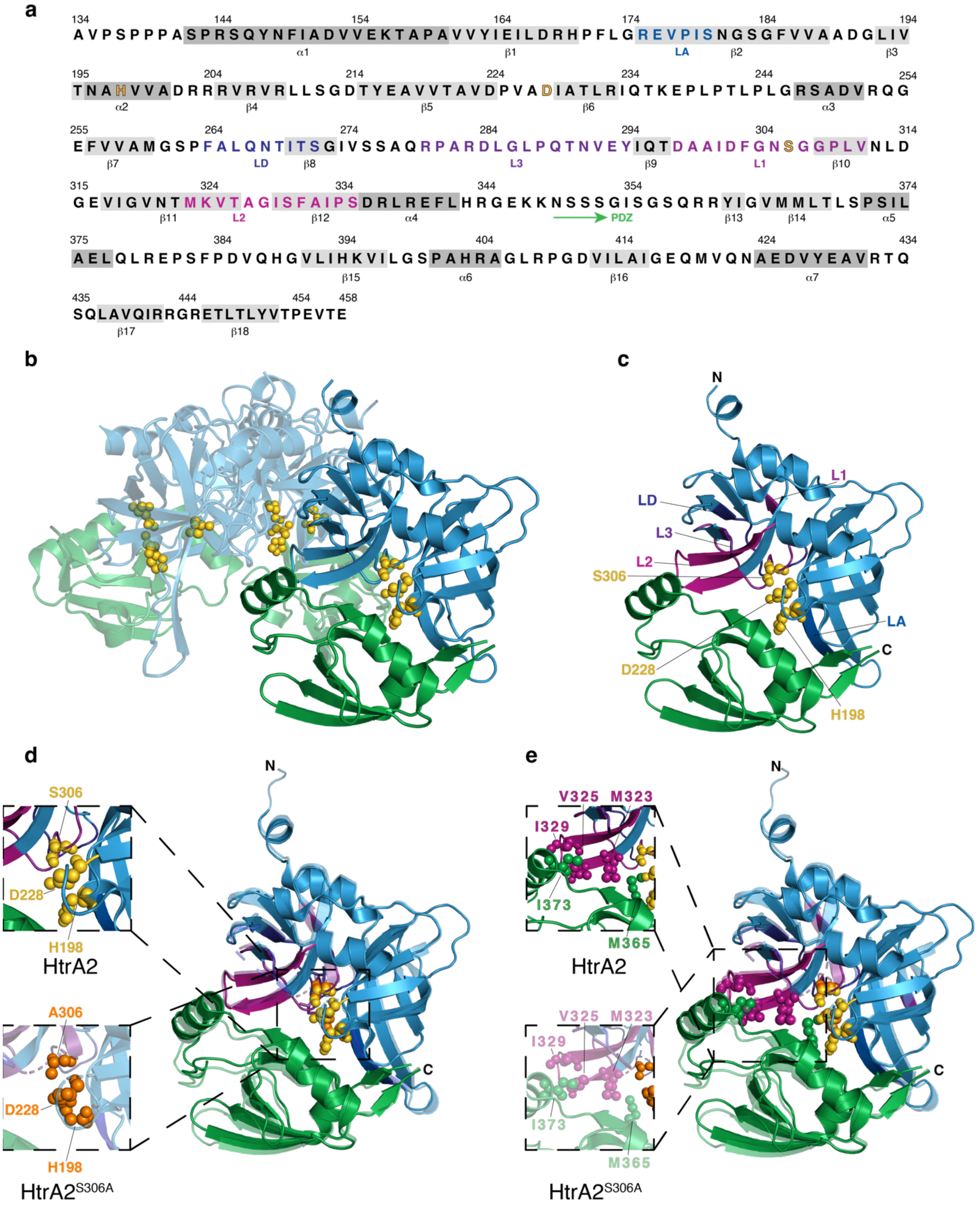


**Supplementary Figure 1. Amino acid sequence and structure of the mature form of human HtrA2 (residues 134-458).**

**a)** Secondary structure elements are indicated as well as the activation loops LA (blue), LD (dark blue), L3 (purple), L1 (magenta) and L2 (pink). Residues H198, D228

and S306 which comprise the active site of HtrA2 are shown in gold. The start of the PDZ-domain is indicated by the green arrow. **b**) Complete trimeric assembly of HtrA2 (PDB-ID: 5M3N) with catalytic residues shown in gold and the individual domains in blue and green, respectively. **c**) Isolated monomer of HtrA2 (PDB-ID: 5M3N) with the regulatory loops and catalytic residues indicated by the color gradient used in panel **a**. **d, e**) Overlay of the catalytic active HtrA2 (PDB-ID: 5M3N) and the catalytically inactive HtrA2$^{S306A}$ (PDB-ID: 1LCY) showing subtle local adaptations besides larger deviations in the active site (**d**) and the PDZ domain protease domain interface (**e**). For a detailed discussion of the differences between the wild-type HtrA2 and the catalytically inactive HtrA2$^{S306A}$ variant the reader is referred to Merski *et al.* [1].

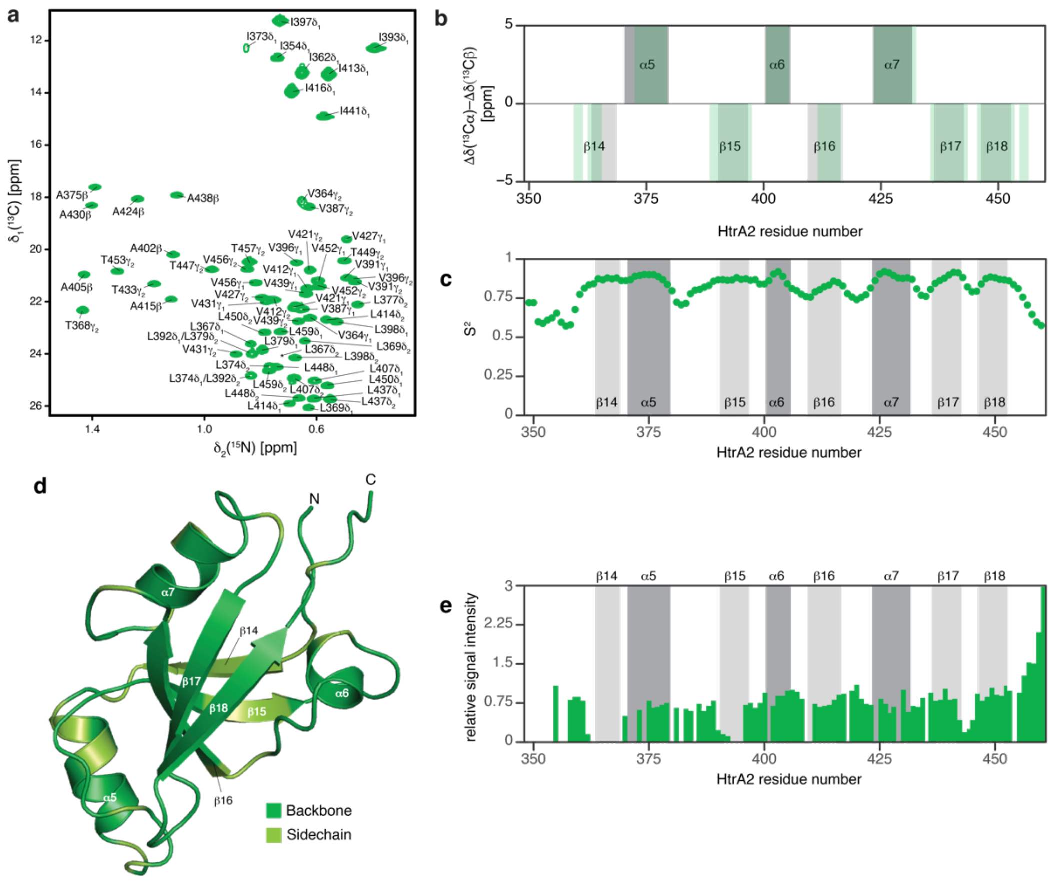


**Supplementary Figure 2. Methyl side chain assignment and chemical shift-based prediction of secondary structure elements.**

**a)** 2D [$^{13}$C,$^{1}$H]-NMR spectrum of ALVIT-labeled-HtrA2-PDZ annotated with the sequence-specific assignments of the methyl side-chain resonances. **b)** Secondary structure elements of the HtrA2-PDZ domain in solution (green) as determined by TALOS-N [2]. The secondary structure elements of the HtrA2-PDZ domain within the full-length crystal structure (PDB-ID:1LCY) are indicated in grey. **c)** The predicted order parameter $S^2$ based on backbone chemical shifts as determined by TALOS-N [2]. **d**) Backbone resonance assignment plotted on the crystal structure of the HtrA2-PDZ domain (PDB-ID: 5M3N). Regions completely assigned by backbone as well as side-chain resonances are shown in green. Regions assigned solely by side-chain resonances are shown in light green. **e**) Relative signal intensity of signals in a 2D [$^{13}$C,$^{1}$H]-NMR spectrum of [*U*-$^{13}$C,$^{15}$N]-HtrA2-PDZ sample. The secondary structure elements of the HtrA2-PDZ domain within the full-length crystal structure (PDB-ID:1LCY) are indicated in grey. Source data are provided as a Source Data File.

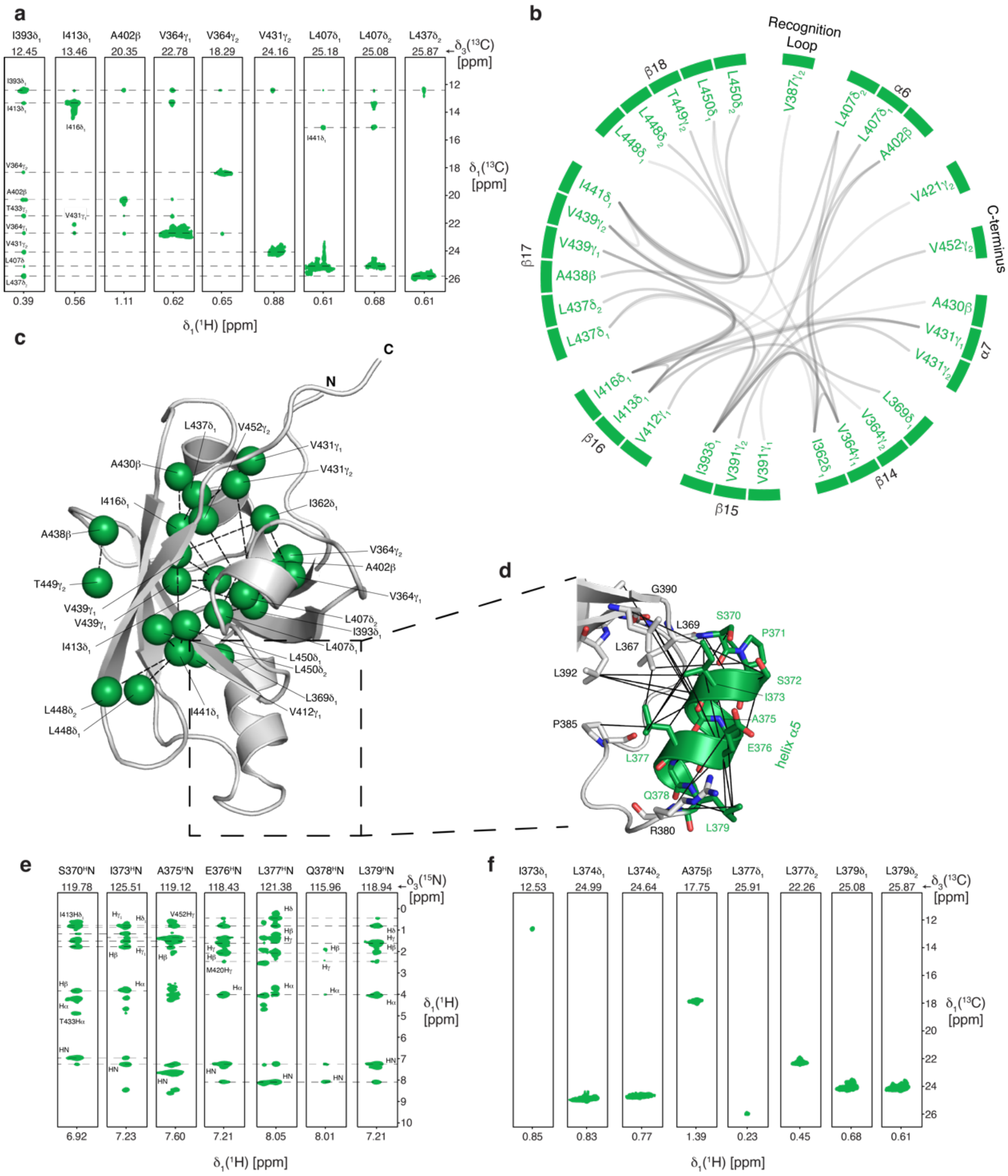


**Supplementary Figure 3. Methyl NOE networks within the HtrA2-PDZ domain.** **a)** Representative NOE strips from a 3D $^{13}C_{methyl}$-$^{13}C_{methyl}$-$^{1}H_{methyl}$ SOFAST NOESY of ALVIT-labeled-HtrA2-PDZ focusing on the NOE network around the I393$\delta_1$ resonance. **b)** Flareplot visualization of the detected inter-methyl NOEs shown in panel **c**, illustrating the connectivity between the individual secondary structure elements. **c)** The detected complete methyl-methyl NOE network within the isolated HtrA2-PDZ-domain plotted on the X-ray structure (PDB-ID: 1LCY). Involved methyl groups are indicated by the green spheres, whereas inter-methyl NOEs are indicated by the broken lines. **d)** Focus on the detected NOEs of the amide groups as well as the amino

acid side-chains within helix $\alpha5$ are consistent with a stable helix fold but show only very few stabilizing interactions with other elements within the PDZ. NOEs are indicated by the broken lines. **e)** Representative NOE strips from a 3D $^{1}H_{all}$-$^{1}H_{amide}$-$^{15}N_{amide}$ NOESY focusing on the NOE network within helix $\alpha5$. **f)** Representative NOE strips from a 3D $^{13}C_{methyl}$-$^{13}C_{methyl}$-$^{1}H_{methyl}$ SOFAST NOESY focusing on the NOE network of methyl bearing amino acids within helix $\alpha5$, indicating the absence of stabilizing long-range interactions.

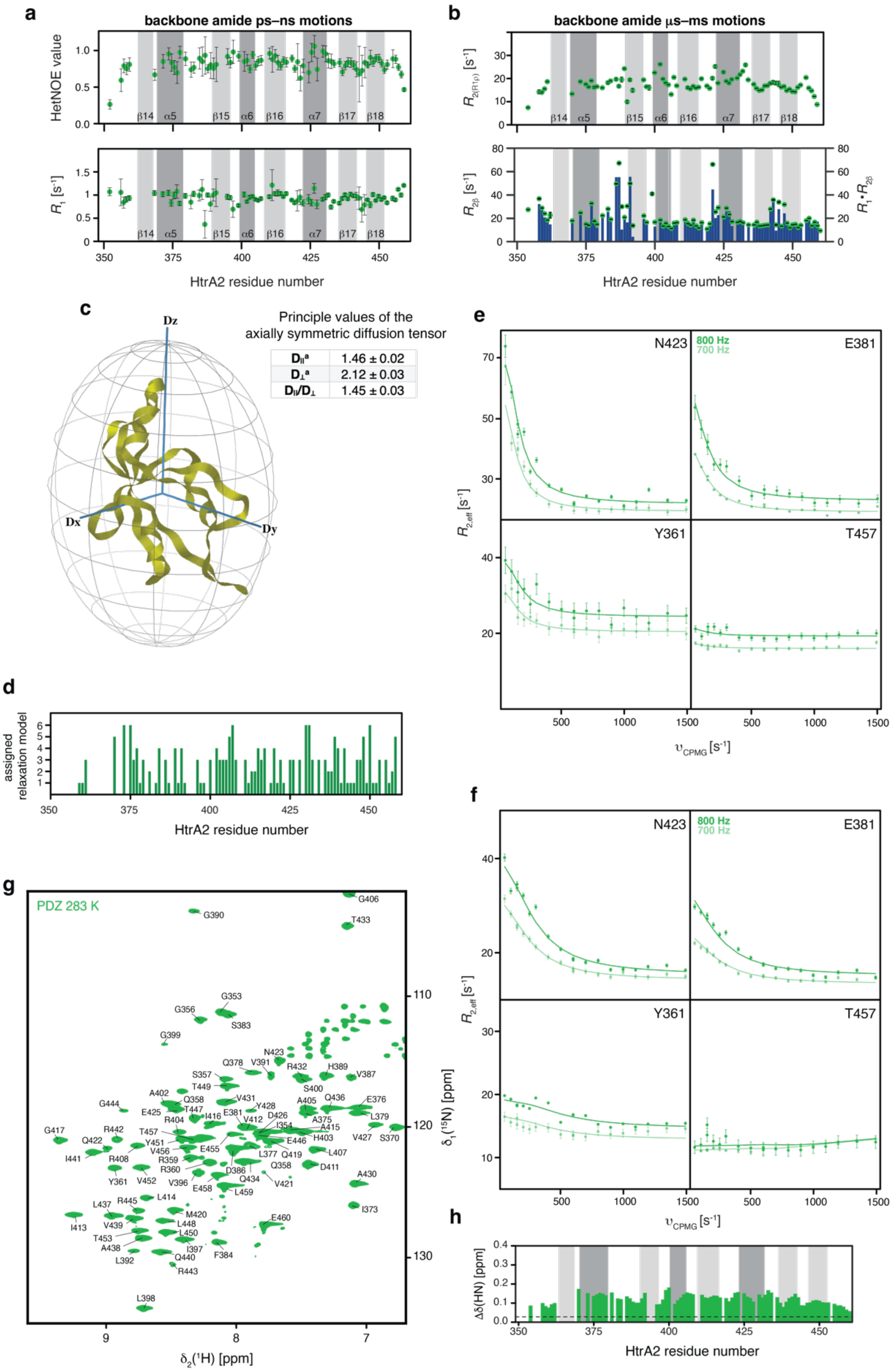


**Supplementary Figure 4. Backbone relaxation data for the HtrA2-PDZ domain.** **a)** Local backbone dynamics on the pico– to nanosecond timescale probed by $^{15}N\{^{1}H\}$-NOE (hetNOE) and $R_1$ measurements. **b**) Obtained transversal relaxation rates ($R_{2\beta}$

and $R_{2(R1\rho)}$) reporting on micro- to millisecond motions. The $R_{2\beta}$ rates are plotted along with the $R_1$•$R_{2\beta}$ values (blue bars). All data are plotted against the HtrA2-PDZ amino acid sequence. Error bars indicate the standard fitting error obtained from the nonlinear least-squares minimization of one experiment (*n* = 1). **c**) Axially symmetric rotational diffusion tensor obtained by the backbone relaxation analysis using Tensor2 [3] employing the HtrA2-PDZ X-ray structure (PDB-ID: 1LCY). The table provides the determined diffusion tensor values in $10^{-7}$ $s^{-1}$. **d**) Residues assigned in the backbone relaxation analysis to the different dynamics model in the Model Free analysis [4,5]. The initial models (models 1–5) were described by Lipari and Szabo [4,5] as well as subsequently extended by Clore and co-workers (model 6) [5]. **e, f)** Exemplary BEST TROSY $^{15}$N CPMG relaxation dispersion profiles at different magnetic field strengths as indicated by the proton frequencies. Data of the same residues is shown at two different temperatures of 283 K (**e**) and 298 K (**f**), respectively. Non-flat profiles indicate the presence of millisecond dynamics. **g**) 2D [$^{15}$N, $^{1}$H]-NMR spectrum of the [*U*-$^{15}$N,$^{13}$C]-HtrA2-PDZ domain at 283 K. The sequence-specific resonance assignment based on triple resonance experiments is indicated. **h**) Basic of the sequence specific resonance assignment to 283 K. CSPs of the amide moieties between the [*U*-$^{15}$N,$^{13}$C]-HtrA2-PDZ domain at 283 K and at 298 K showing similar temperature dependence over the visible signals facilitating the direct transfer of the assignment. Source data are provided as a Source Data File.

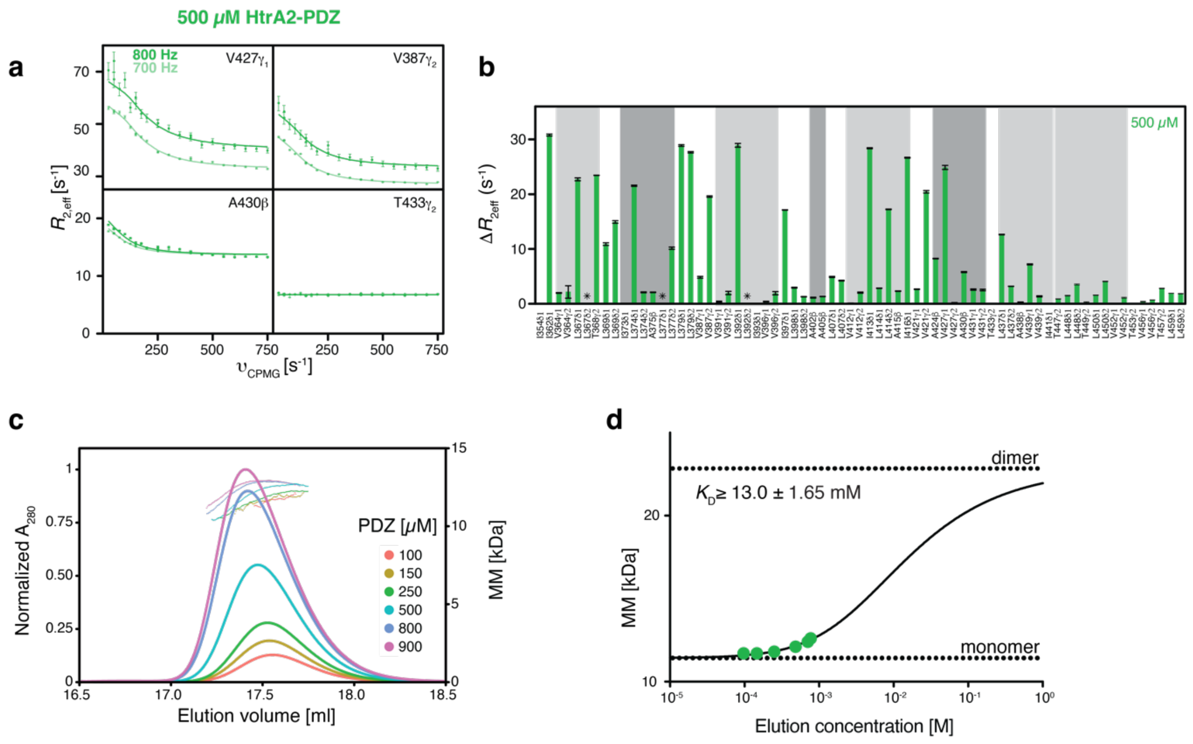


**Supplementary Figure 5. Side chain relaxation and SEC MALS analysis of the HtrA2-PDZ domain.**

**a)** Exemplary $^{13}$C methyl MQ CPMG relaxation dispersion profiles at different magnetic field strengths as indicated by the respective proton frequencies. Data for a 500 µM ALVIT-PDZ sample are shown. Non-flat profiles indicate millisecond dynamics. **b)** $\Delta R_{2eff}$ values for the ALVIT methyl-groups, obtained from the difference of $R_{2eff}$ at the lowest and highest CPMG frequency $\upsilon_{CPMG}$. Error bars indicate the standard fitting error obtained from the nonlinear least-squares minimization of one experiment ($n$ = 1). **c)** SEC elution profiles plotted as normalized absorbance ($A_{280}$) (solid lines, left axis) and MALS apparent molecular mass (dotted lines, right axis) as a function of the indicated protein concentrations. **d)** Nonlinear regression fit of the averaged molar mass as a function of the elution concentration to a monomer-dimer equilibrium. Dotted lines represent the theoretical monomer and dimer molecular masses, respectively. Estimated molecular weights are reported in **Supplementary Table 1.** Source data are provided as a Source Data File.

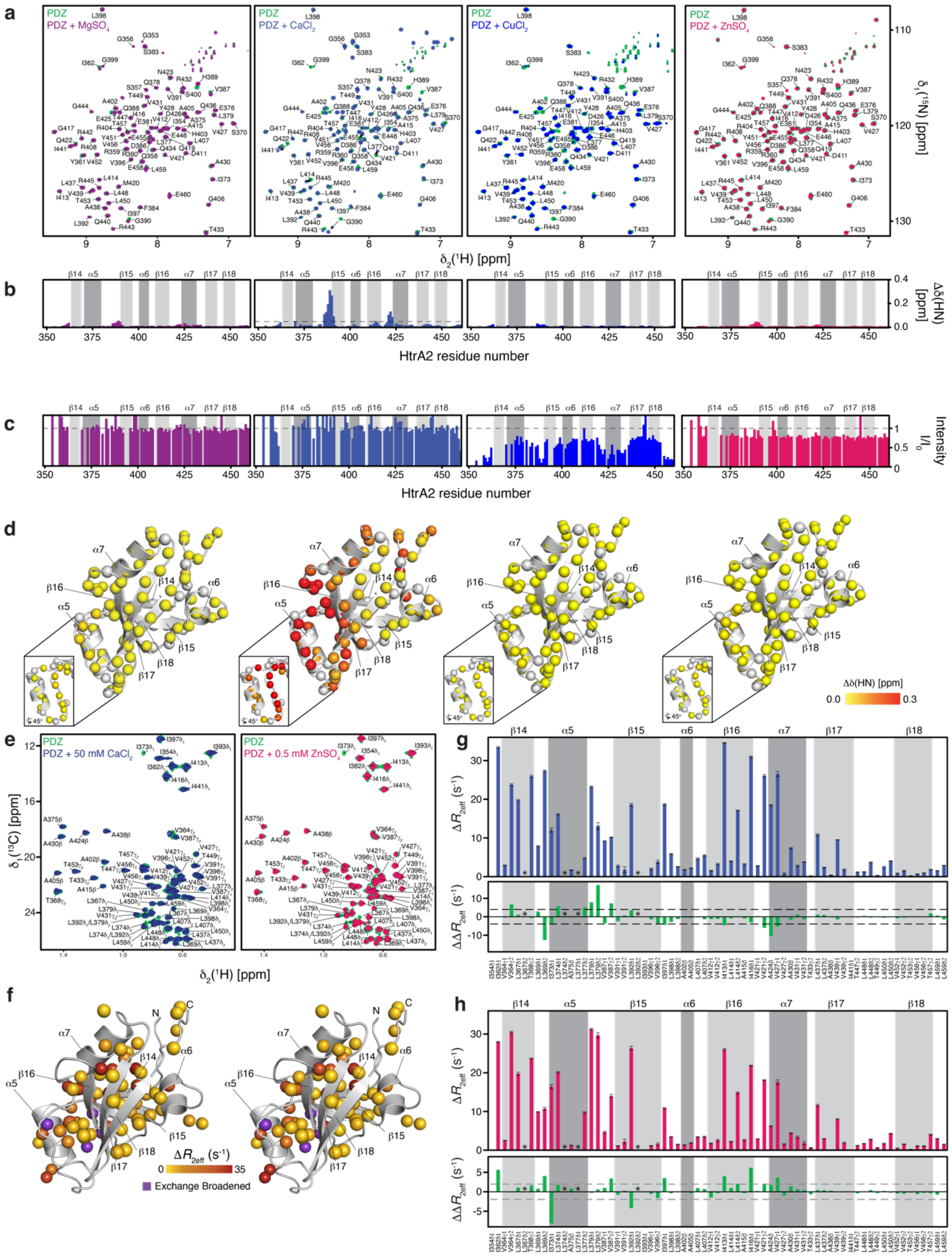


**Supplementary Figure 6. Spectral changes upon addition of divalent cations.**

**a)** 2D [$^{15}$N, $^{1}$H]-NMR spectrum of the 200 µM [*U*-$^{15}$N]-HtrA2-PDZ domain (green) and after addition of 25 mM $MgSO_4$ (purple), 25 mM $CaCl_2$ (dark blue), 2 mM $CuCl_2$ (blue), and 2 mM $ZnSO_4$ (magenta). The sequence-specific resonance assignment based on triple resonance experiments is indicated. **b, c)** CSPs (**b**) and signal attenuations (**c**) of the amide moiety upon interaction with the different divalent cations as indicated in

panel **a**. The dotted line indicates a significance level of one S.D. **d)** Perturbed resonances are mapped on the crystal structure of the HtrA2-PDZ (PDB-ID: 1LCY). The extent of the detected chemical shift perturbations (CSPs) upon addition of the different divalent cations is indicated by the yellow to red gradient. **e)** 2D [$^{13}C$, $^{1}H$]-NMR spectrum of the 500 µM ALVIT methyl-group labelled HtrA2-PDZ domain (green) and after addition of 50 mM $CaCl_2$ (dark blue, left) or 0.5 mM $ZnSO_4$ (magenta, right). The sequence-specific resonance assignment of the methyl groups is indicated. **f**) Structural view of the amplitude of the CPMG relaxation dispersion profiles $\Delta R_{2eff}$ at 16.4 T of the 500 µM ALVIT–PDZ sample at 298 K in the presence of 50 mM $CaCl_2$ (left) or 0.5 mM $ZnSO_4$ (right). **g)** $\Delta R_{2eff}$ values for the ALVIT methyl-groups in the presence of 50 mM $CaCl_2$ (dark blue), obtained from the difference of $R_{2eff}$ at the lowest and highest CPMG frequency $\upsilon_{CPMG}$ (top). $\Delta\Delta R_{2eff}$ represents the difference of the data in presence and absence of $CaCl_2$ (green, bottom). **h)** $\Delta R_{2eff}$ values for the ALVIT methyl-groups in the presence of 0.5 mM $ZnSO_4$ (magenta), obtained from the difference of $R_{2eff}$ at the lowest and highest CPMG frequency $\upsilon_{CPMG}$ (top). $\Delta\Delta R_{2eff}$ represents the difference of the data in presence and absence of $ZnSO_4$ (green, bottom). Error bars (panel **g** and **h**) indicate the standard fitting error obtained from the nonlinear least-squares minimization of one experiment (*n* = 1). Source data are provided as a Source Data File.

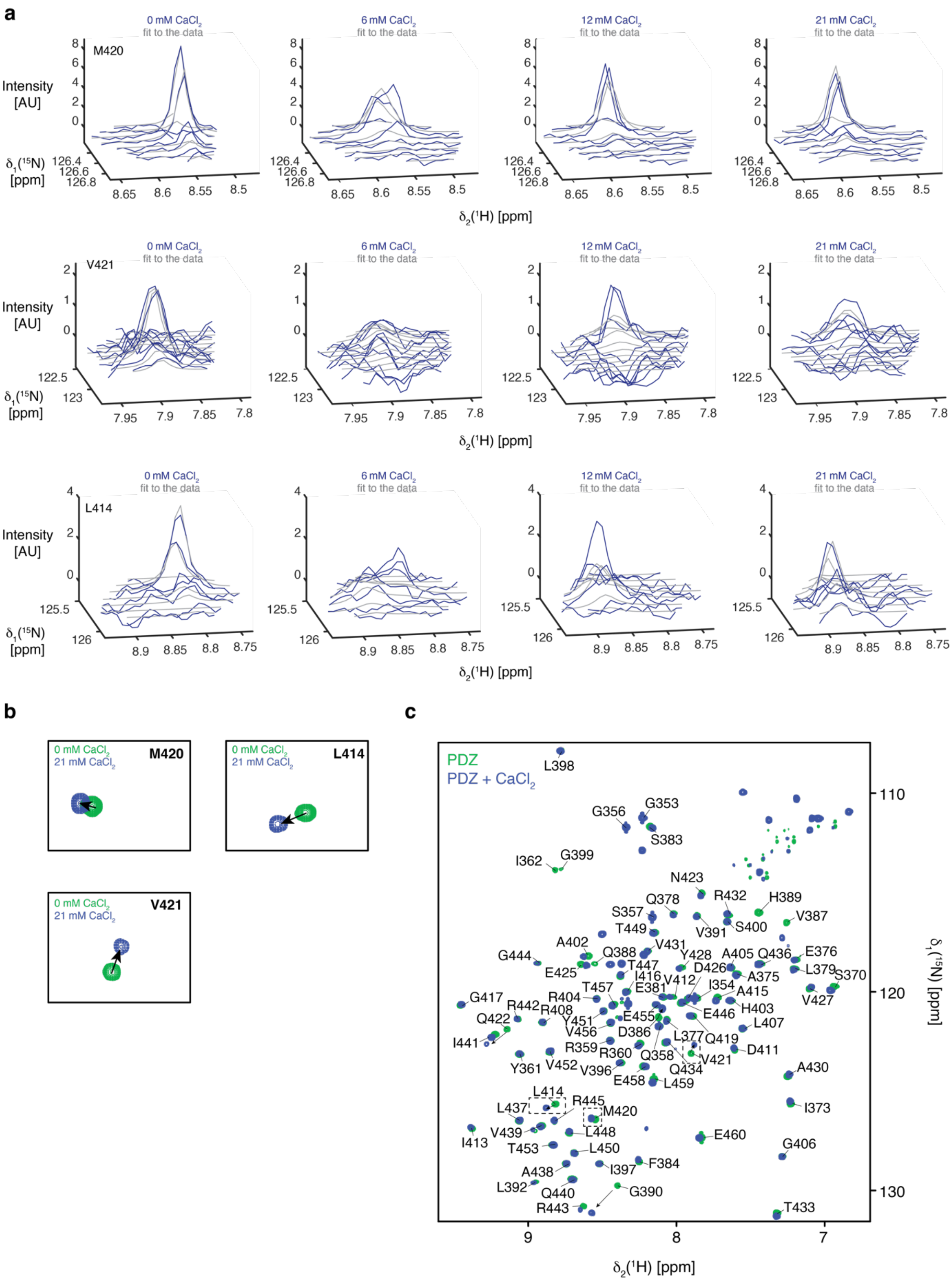


**Supplementary Figure 7. a)** Global analysis of the two-dimensional line-shapes was performed with the program TITAN [7] to extract apparent thermodynamic constants. The quality of the 2D line shape analysis was assessed by comparing the experimental data (dark blue) for the individual titration steps with the obtained fitted values (grey).

**b**) Selected resonances of the 2D [$^{15}$N, $^{1}$H]-NMR spectrum shown in panel **c** are shown as a zoom. **c**) 2D [$^{15}$N, $^{1}$H]-NMR spectrum of the 200 μM [*U*-$^{15}$N]-HtrA2-PDZ domain (green) and after addition of 25 mM $CaCl_2$ (dark blue) sequence-specific resonance assignment based on triple resonance experiments is indicated.

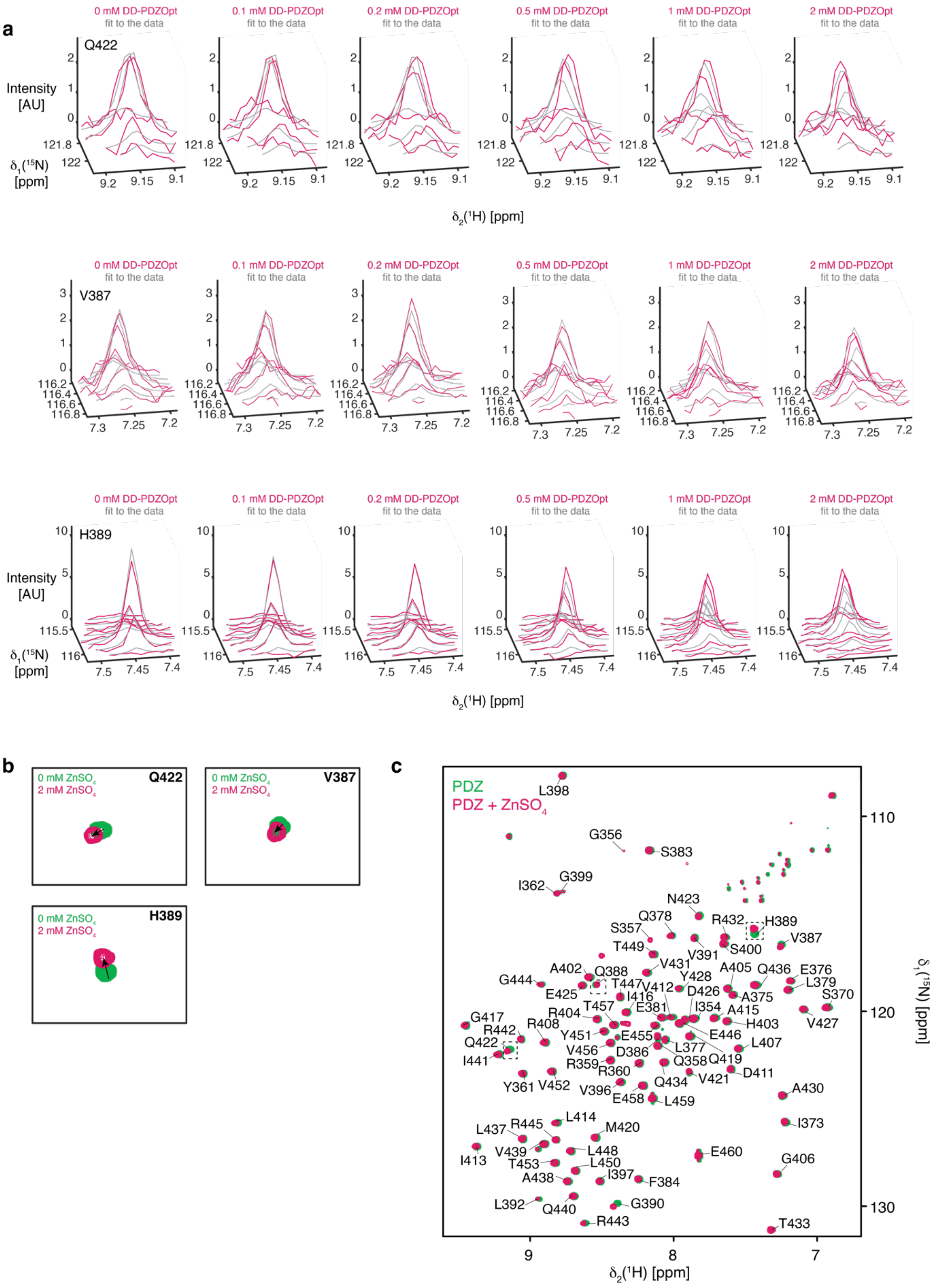

**Supplementary Figure 8. a)** Global analysis of the two-dimensional line-shapes was performed with the program TITAN [7] to extract apparent thermodynamic constants. The quality of the 2D line shape analysis was assessed by comparing the experimental

data (magenta) for the individual titration steps with the obtained fitted values (grey). **b**) Selected resonances of the 2D [$^{15}$N, $^{1}$H]-NMR spectrum shown in panel **c** are shown as a zoom. **c**) 2D [$^{15}$N, $^{1}$H]-NMR spectrum of the 200 µM [*U*-$^{15}$N]-HtrA2-PDZ domain (green) and after addition of 2 mM $ZnSO_4$ (magenta) sequence-specific resonance assignment based on triple resonance experiments is indicated.

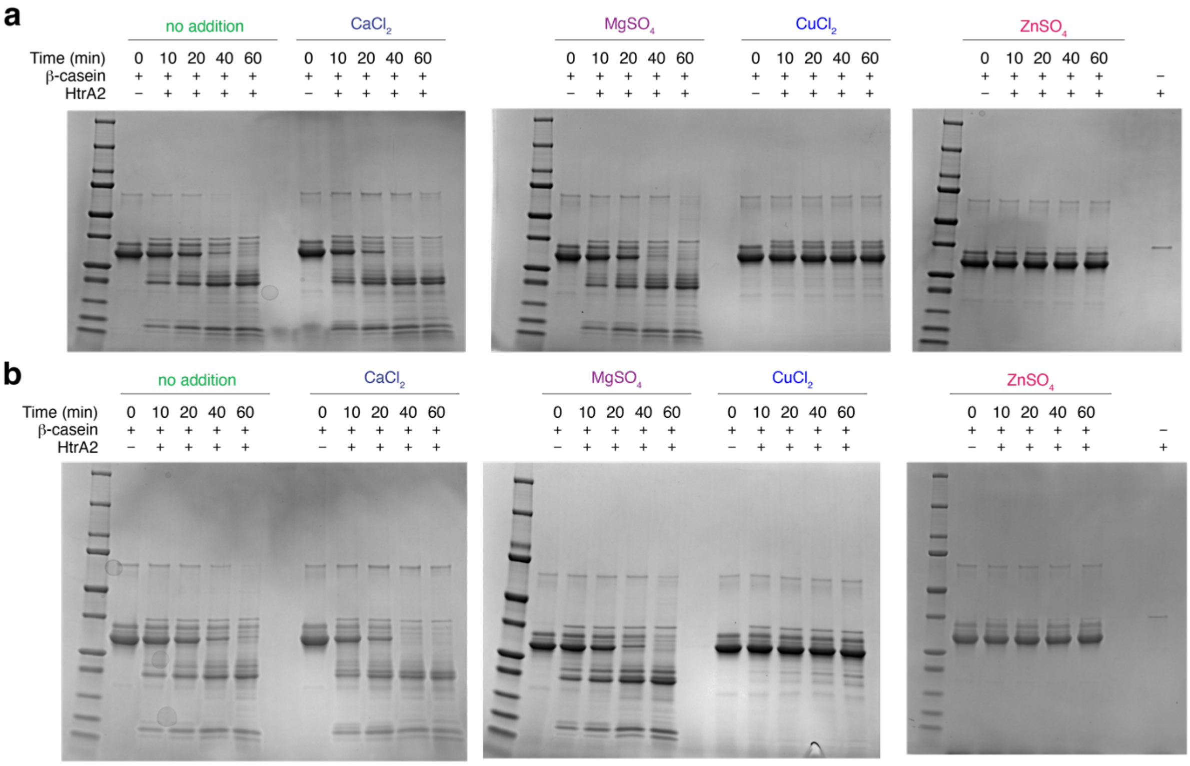


**Supplementary Figure 9. Modulation of HtrA2 proteolytic activity by divalent cations.**

**a, b)** Cleavage assay with wild-type HtrA2 using β-casein as a substrate in buffer supplemented with the indicated divalent cations in comparison to buffer without metal supplementation. Gels in panel **a** and **b** show the results of repeat assays of the cleavage assay depicted in **Fig. 3a**.

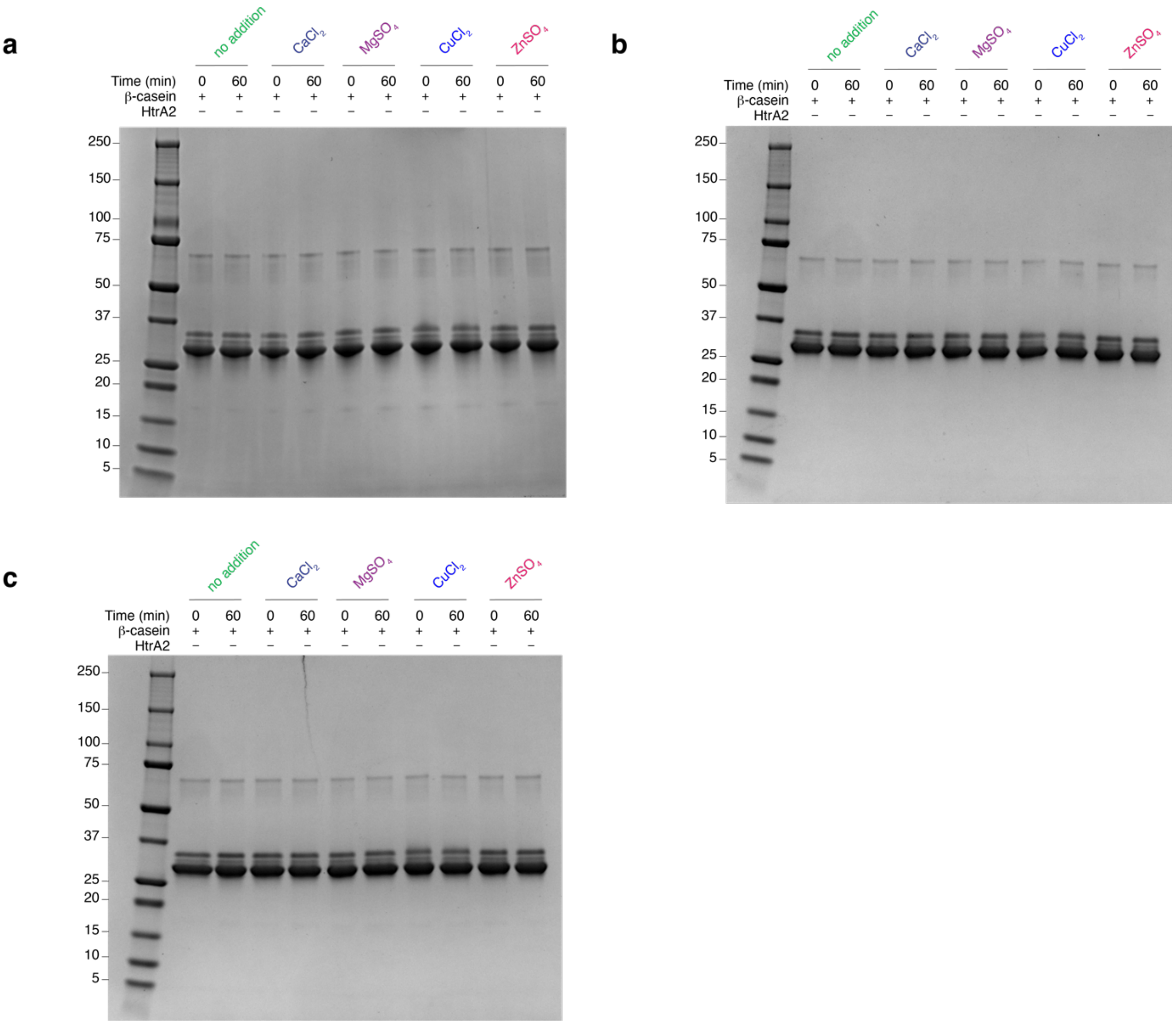


**Supplementary Figure 10. β-casein cleavage assay controls.**

**a–c)** Triplicate controls of β-casein in standard cleavage assay buffer with or without addition of 2 mM of the indicated metal ions and in absence of HtrA2.

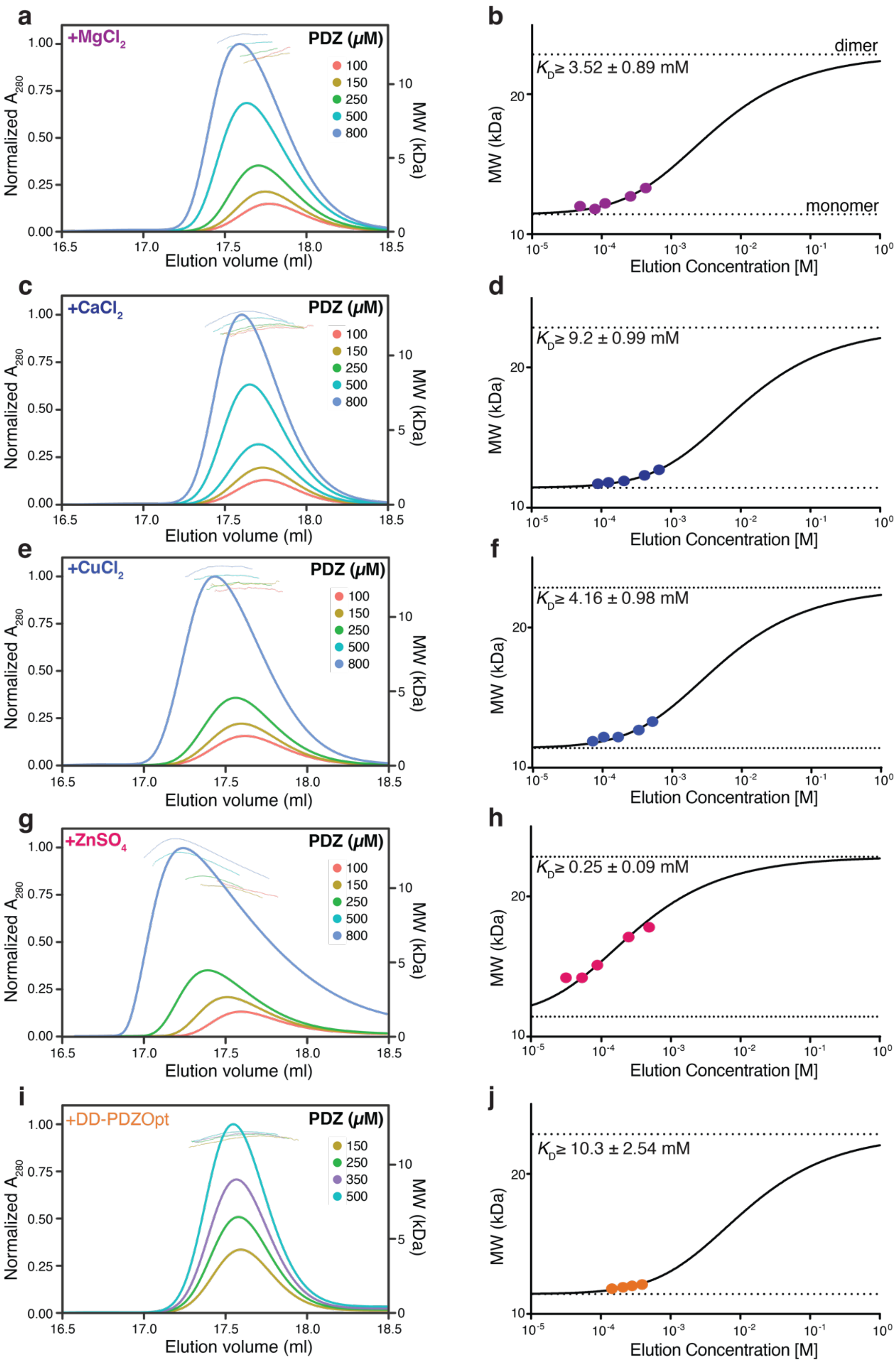


**Supplementary Figure 11. Influence of divalent cations and the activating peptide DD-PDZOpt on PDZ dimerization.**

**a, c, e, g, i)** SEC elution profiles plotted as normalized absorbance ($A_{280}$) (solid lines, left axis) and MALS apparent molecular mass (dotted lines, right axis) as a function of the indicated protein concentrations and the addition of the indicated divalent cations or the DD-PDZOpt peptide. **b, d, f, h, j)** Nonlinear regression fit of the averaged molar mass as a function of the elution concentration to a monomer-dimer equilibrium. Dotted lines represent the theoretical monomer and dimer molecular masses, respectively. Estimated molecular weights are reported in **Supplementary Table 1.** Source data are provided as a Source Data File.

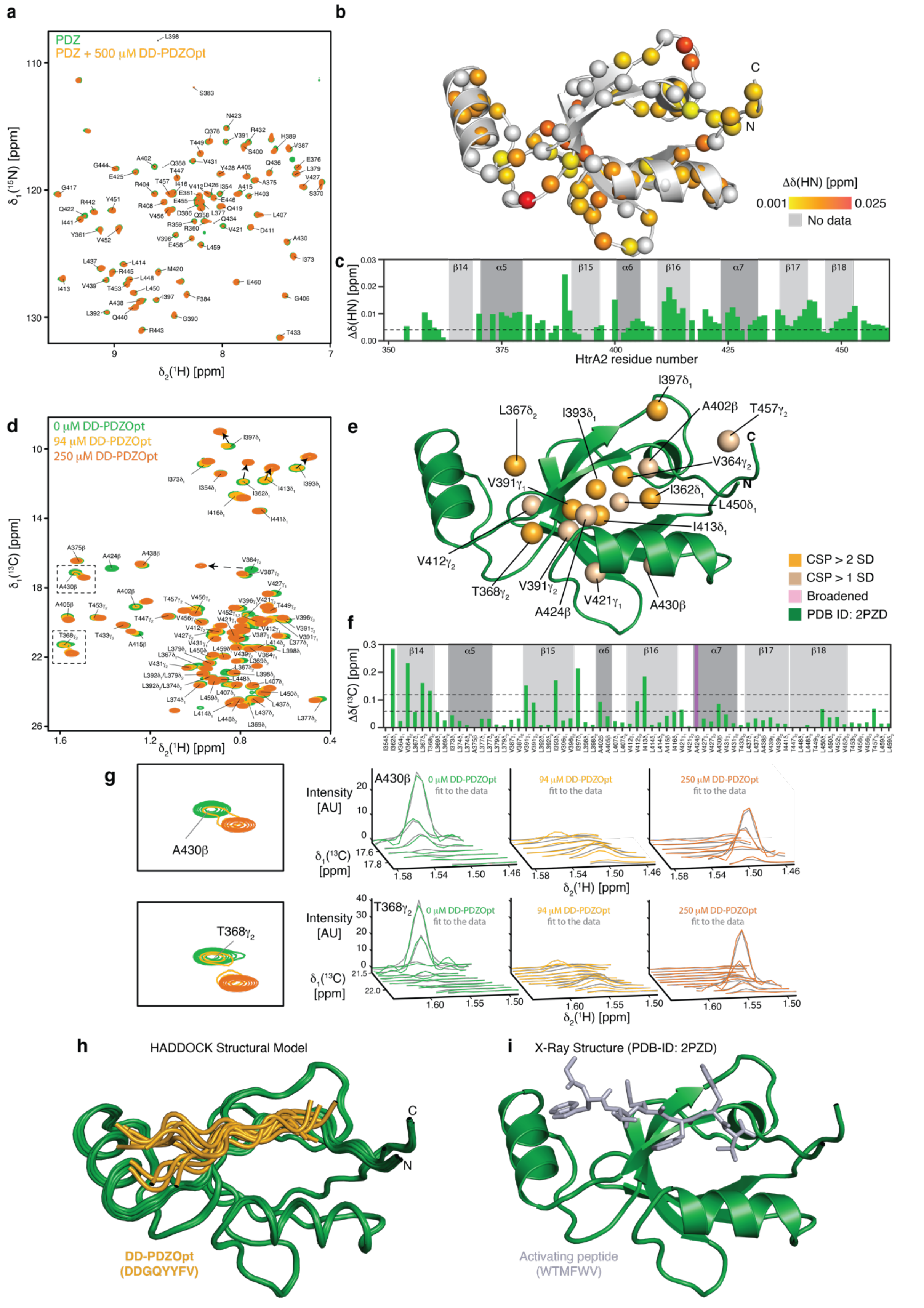


**Supplementary Figure 12. Interaction of the PDZ-domain with an activating peptide.**

**a)** 2D [$^{15}$N, $^{1}$H]-NMR spectrum of 150 µM [*U*-$^{15}$N,$^{13}$C]-HtrA2-PDZ (green) and after addition of 500 µM DD-PDZOpt peptide (orange). The sequence-specific resonance assignment based on triple resonance experiments for the peptide-free state is indicated. **b)** Perturbed resonances are mapped on the crystal structure of the HtrA2-PDZ in complex with an activating peptide (PDB-ID: 2PZD). The extent of the detected chemical shift perturbations (CSPs) is indicated by the yellow to red gradient. **c)** CSPs of the amide moiety upon binding to the DD-PDZOpt peptide. The dotted line indicates a significance level of one S.D. Source data are provided as a Source Data File. **d)** 2D [$^{13}$C, $^{1}$H]-NMR spectrum of 150 µM ALVIT-methyl group labelled HtrA2-PDZ domain (green) and after addition of 94 µM DD-PDZOpt peptide (yellow), and of 250 µM DD-PDZOpt peptide (orange). The sequence-specific resonance assignment of the methyl groups for the peptide-free state is indicated. Arrows indicate large CSPs. **e)** Perturbed methyl resonances are mapped on the crystal structure of the HtrA2-PDZ in complex with an activating peptide (PDB-ID: 2PZD). The extent of the detected chemical shift perturbations (CSPs) is indicated by the indicated colors for shifts larger than one S.D., two S.D. as well as broadened resonances. **f)** CSPs of the methyl group resonances upon binding to 250 µM of the DD-PDZOpt peptide. The dotted lines indicate a significance level of one and two S.D., respectively. Source data are provided as a Source Data File. **g)** Global analysis of the two-dimensional line-shapes was performed with the program TITAN [7] to extract apparent thermodynamic constants. Selected resonances (A430β and T368$\gamma_2$) of the 2D [$^{13}$C, $^{1}$H]-NMR spectrum shown in panel **d** are shown as a zoom (left). The quality of the 2D line shape analysis was assessed by comparing the experimental data (green, yellow, orange) for the individual titration steps with the obtained fitted values (grey). **h)** Structural ensemble of the ten lowest energy structures of the top cluster obtained with HADDOCK docking [8] based on chemical shift perturbation data. HtrA2-PDZ is depicted in green and the DD-PDZOpt peptide in yellow. **i)** X-ray structure of the HtrA2-PDZ (green) and an activator peptide (WTMFWV: silver) (PDB-ID: 2PZD).

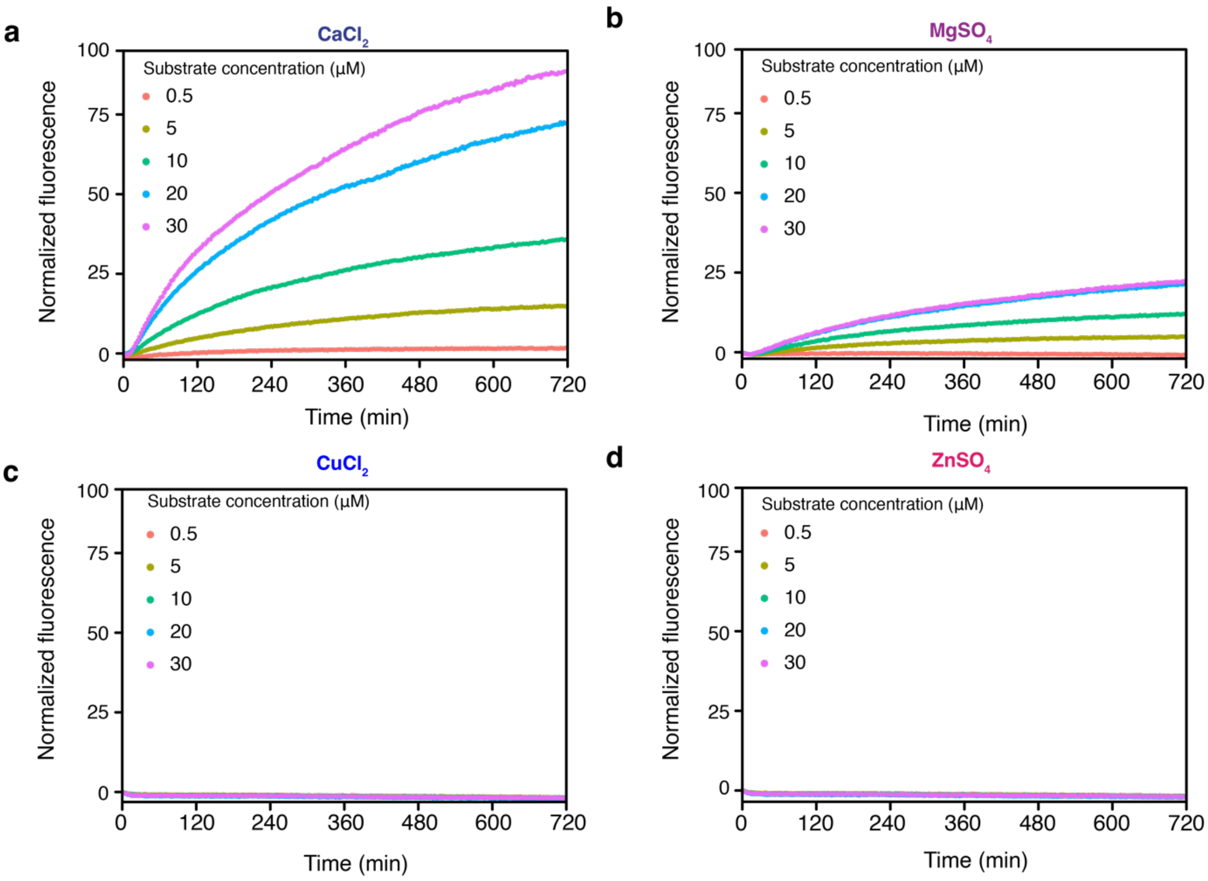


**Supplementary Figure 13. Fluorescence cleavage assays employing divalent metal ion supplementation with varied substrate concentration.**

**a–d)** Normalized fluorescence cleavage assay data showing the activity of wild-type HtrA2 together with a fixed concentration of activating peptide (DD-PDZOpt) at 50 μM, together with varying concentrations of an optimized substrate peptide (H2-Opt) in supplemented with 2 mM $CaCl_2$ (**a**), 2mM $MgSO_4$ (**b**), 2 mM $CuCl_2$ (**c**) or 2 mM $ZnSO_4$ (**d**). The assay was done as three repeat measurements, and the raw data represents the average of the three measurements. The experimental data was analyzed using InterferENZY [9] as outlined in the methods section and the resulting kinetic parameters are listed in **Supplementary Table 3**.

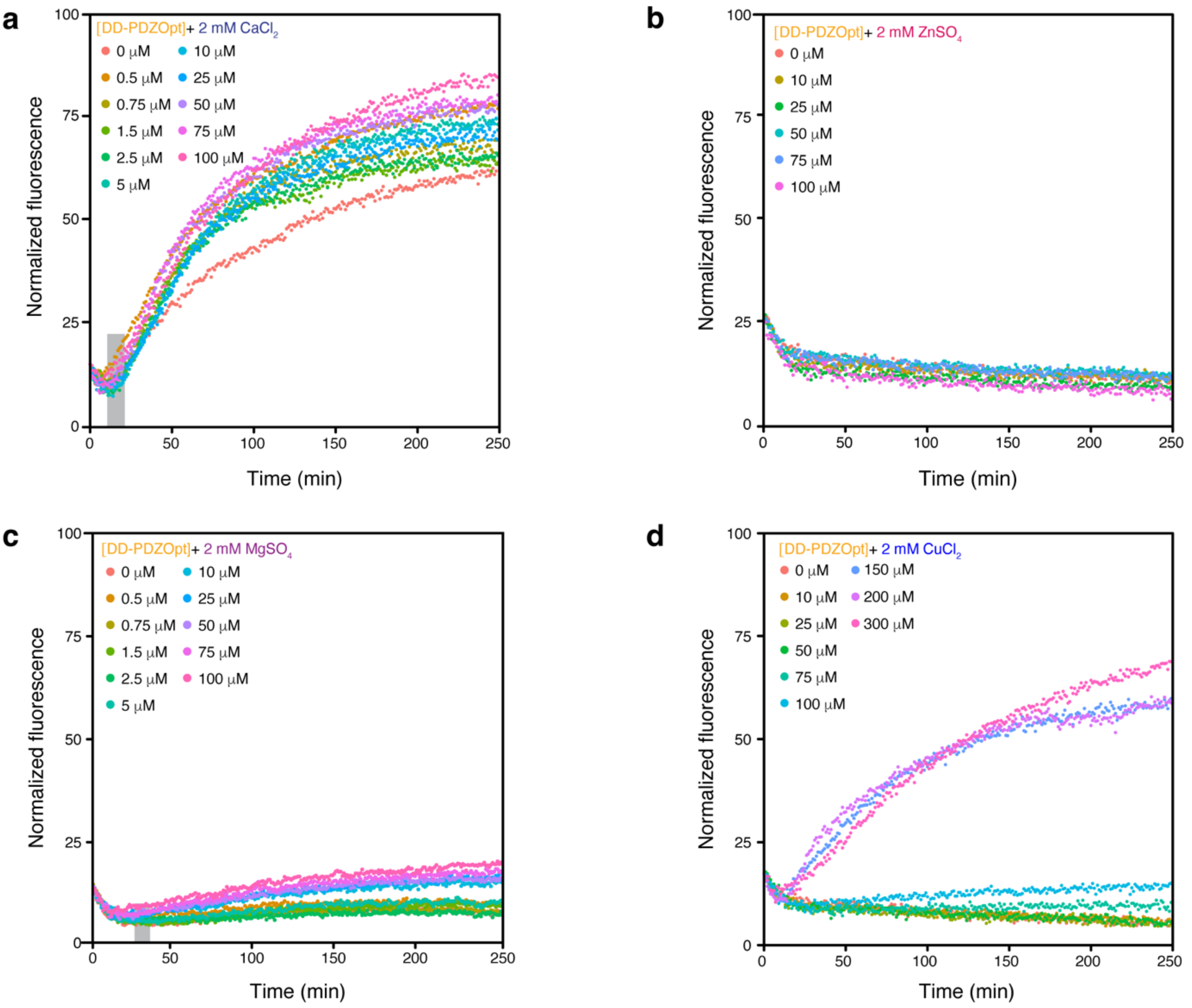


**Supplementary Figure 14. Fluorescence cleavage assays employing divalent metal ion supplementation with varied activating peptide concentration.**

**a–d)** Normalized fluorescence cleavage assay data showing the activity of wild-type HtrA2 towards an optimized substrate (H2-Opt) at a fixed concentration of 20 μM together with varying concentrations of activating peptide (DD-PDZOpt) in buffer supplemented with 2 mM $CaCl_2$ (**a**), 2 mM $ZnSO_4$ (**b**), 2mM $MgSO_4$ (**c**), and 2 mM $CuCl_2$ (**d**). The grey shaded area indicates the region analyzed for extracting the velocity.

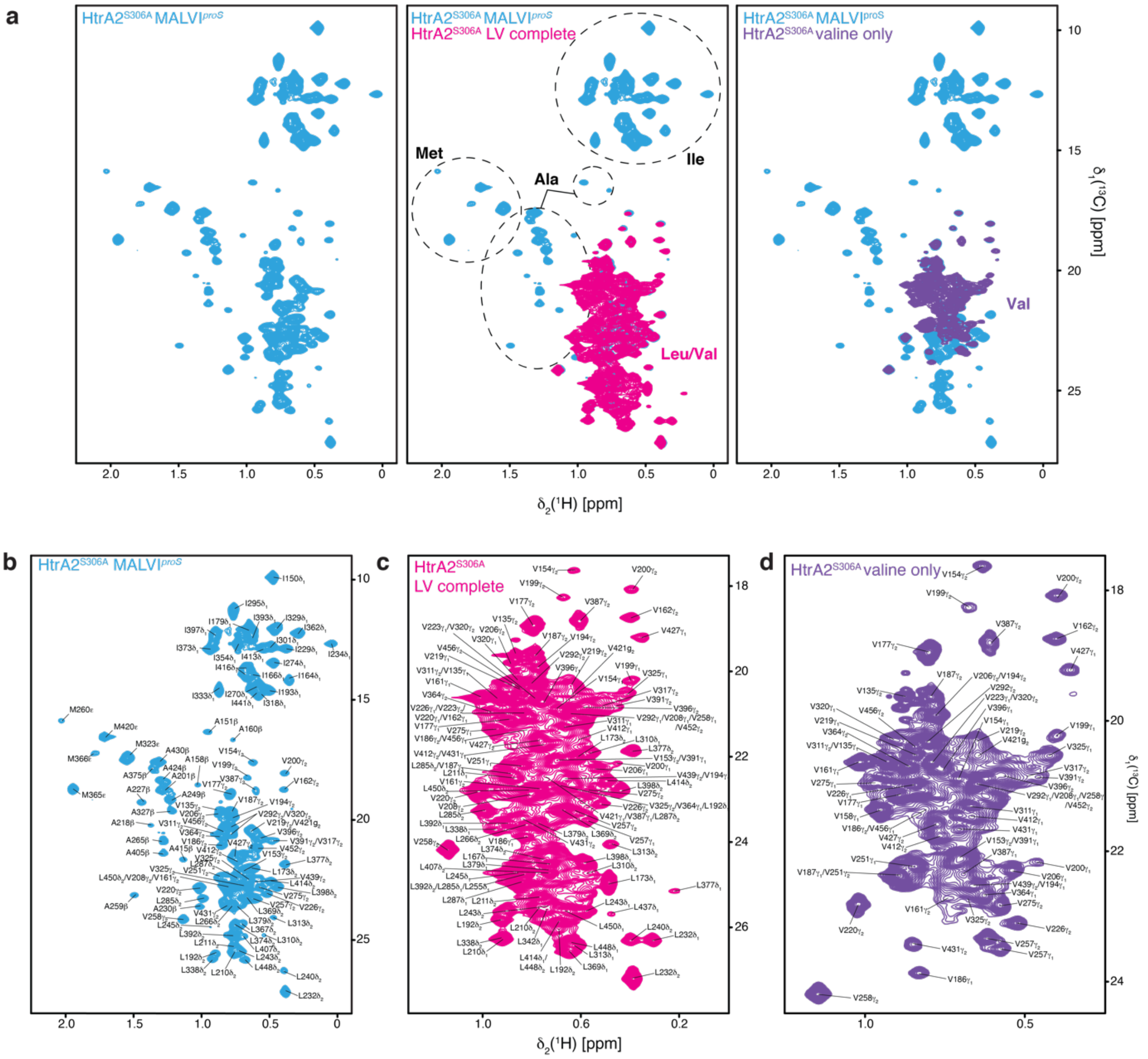


**Supplementary Figure 15. Methyl group assignment of full-length HtrA2$^{S306A}$.** **a)** $[^{13}C,^{1}H]$-NMR spectra of HtrA2$^{S306A}$ MALVI$^{proS}$ (blue), LV (leucine, valine)-(magenta), or V (valine) only (purple) labeled samples enabled, together with previously reported methyl group assignments for an HtrA2$^{S306A}$ ILVM$^{proR}$ sample by Toyama *et al.,* [10] complete amino acid typing of the observed methyl resonances of the MALVI$^{proS}$ sample. **b–d)** NOE and HMBC-HMQC-based sequence-specific resonance assignment of the MALVI$^{proS}$ (**b**), the LV- (**c**) as well as the valine only sample (**d**).

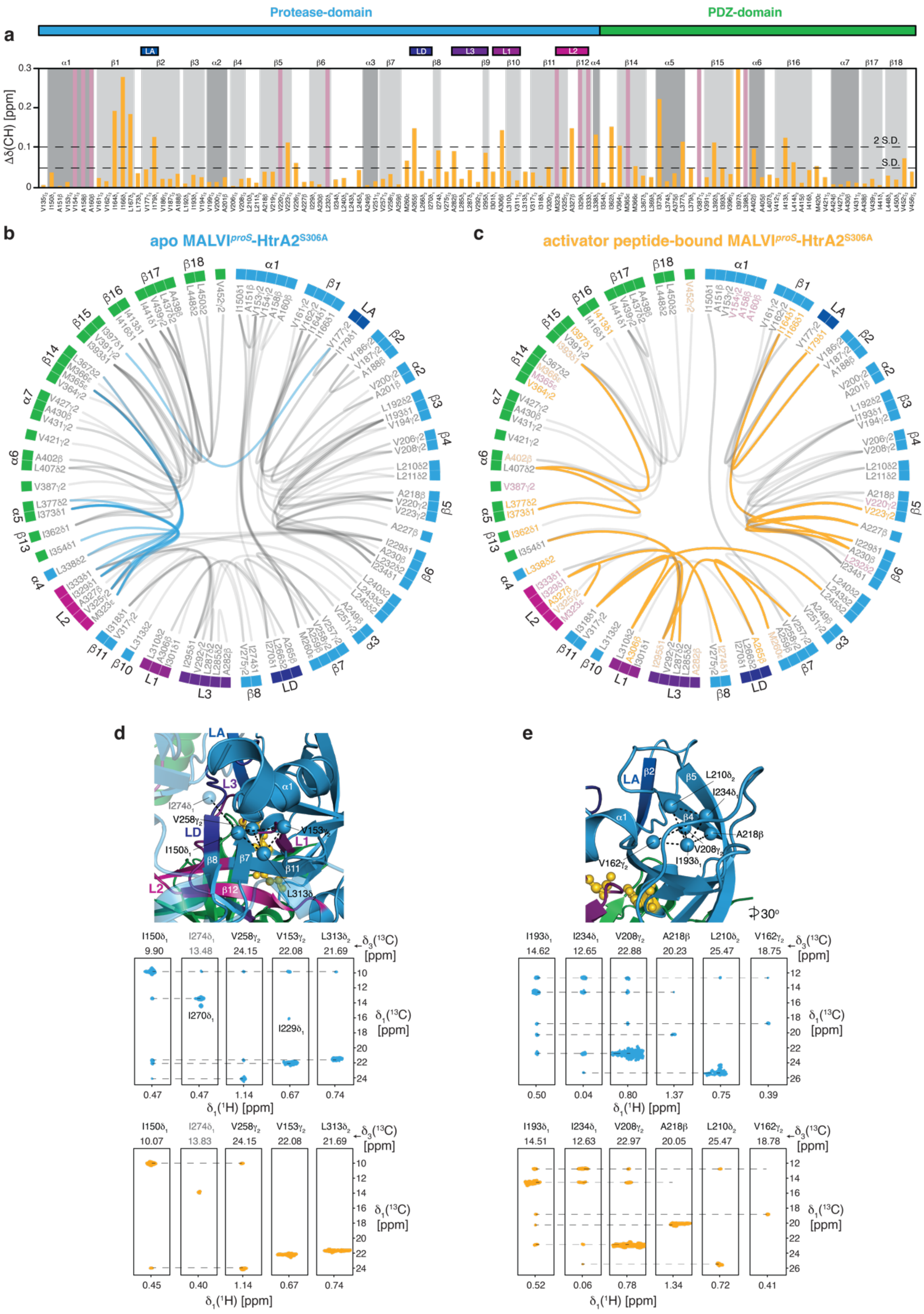


**Supplementary Fig.16. Allosteric NOE network of HtrA2$^{S306A}$.**

**a)** CSPs observed upon addition of 5 equivalents of DD-PDZOpt to MALVI$^{proS}$-HtrA2$^{S306A}$. Resonances broadened beyond detection are indicated by purple bars. The individual domains of HtrA2 are indicated in blue (protease domain) and green (PDZ domain), respectively. Source data are provided as a Source Data File. **b)** Flareplot visualization of the detected inter-methyl NOEs for full-length apo MALVI$^{proS}$-HtrA2$^{S306A}$ showing the connectivity between the individual secondary structure elements of the different domains (protease domain (blue), the regulatory loops (magenta, purple, dark blue) and the PDZ domain (green)) in the absence of activating peptide. Connectivities highlighted in blue indicate interdomain contacts stabilizing the closed apo state. **c)** Flareplot visualization of the detected inter-methyl NOEs for full-length DD-PDZOpt peptide-bound MALVI$^{proS}$-HtrA2$^{S306A}$ showing the connectivity between the individual secondary structure elements of the different domains (protease domain (blue), the regulatory loops (magenta, purple, dark blue) and the PDZ domain (green)) in the presence of the activating peptide. Connectivities highlighted in yellow indicate NOE contacts only observed in the presence of the activating peptide pointing to local structural changes underlying HtrA2 activation. **d, e**) Representative methyl NOE clusters (top) and the respective strips from a 3D $^{13}C_{methyl}$-$^{13}C_{methyl}$-$^{1}H_{methyl}$ SOFAST NOESY experiments of MALVI$^{proS}$-HtrA2$^{S306A}$ (blue) and activating peptide bound MALVI$^{proS}$-HtrA2$^{S306A}$ (yellow). Participating methyl groups are indicated in black if there are from the same protomer whereas in grey if they are from an adjacent HtrA2 protomer. Positions of secondary structure elements and regulatory loops are indicated. Inter-methyl NOEs are highlighted by the broken lines. The orientation of the top panel in **e** relative to panel **d** is indicated.

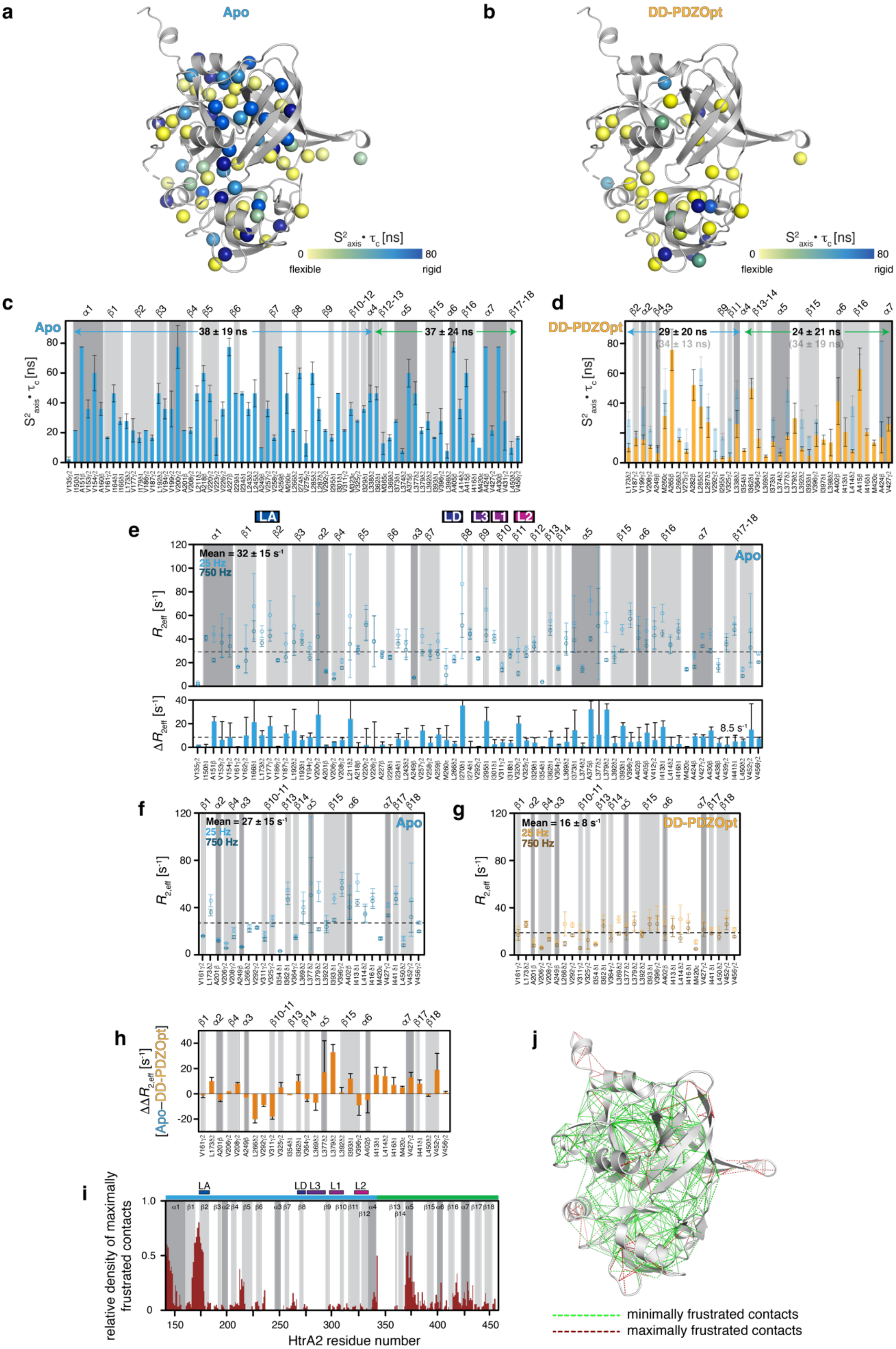

a
Apo
$S^2_{axis} \cdot \tau_c$ [ns]
0
80
flexible
rigid
b
DD-PDZOpt
c
Apo
38 ± 19 ns
37 ± 24 ns
$S^2_{axis} \cdot \tau_c$ [ns]
d
DD-PDZOpt
29 ± 20 ns
(34 ± 13 ns)
24 ± 21 ns
(34 ± 19 ns)
e
LA
LD
L3
L1
L2
Mean = 32 ± 15 s⁻¹
25 Hz
750 Hz
Apo
$R_{2eff}$ [s⁻¹]
$\Delta R_{2eff}$ [s⁻¹]
8.5 s⁻¹
f
Mean = 27 ± 15 s⁻¹
25 Hz
750 Hz
Apo
g
Mean = 16 ± 8 s⁻¹
25 Hz
750 Hz
DD-PDZOpt
h
$\Delta\Delta R_{2eff}$ [s⁻¹]
[Apo–DD-PDZOpt]
i
relative density of maximally frustrated contacts
HtrA2 residue number
j
minimally frustrated contacts
maximally frustrated contacts

**Supplementary Fig. 17. Methyl group dynamic analysis of the HtrA2$^{S306A}$ inactive apo state and the DD-PDZOpt bound activated state**.

**a)** Local methyl-group dynamics on the pico- to nanosecond timescale of the apo state of HtrA2$^{S306A}$ probed by methyl single-quantum (SQ) and triple-quantum (TQ) relaxation experiments showing the product of the local order parameter and the overall tumbling constant, $S^2_{axis}\bullet\tau_C$. Measurements were performed on a 100 µM MALVI$^{proS}$-HtrA2$^{S306A}$ sample at 313 K. The methyl-groups are shown as spheres and the obtained $S^2_{axis}\bullet\tau_C$-values by the yellow to blue gradient. **b)** Local methyl-group dynamics on the pico- to nanosecond timescale of the DD-PDZOpt bound state of HtrA2$^{S306A}$ probed by methyl single-quantum (SQ) and triple-quantum (TQ) relaxation experiments showing the product of the local order parameter and the overall tumbling constant, $S^2_{axis}\bullet\tau_C$. Measurements were performed on a 100 µM MALVI$^{proS}$-HtrA2$^{S306A}$ sample at 313K with 520 µM DD-PDZOpt. The methyl-groups are shown as spheres and the obtained $S^2_{axis}\bullet\tau_C$-values by the yellow to blue gradient. **c**) $S^2_{axis}\bullet\tau_C$-values of apo HtrA2$^{S306A}$ plotted against the HtrA2 amino acid sequence. Secondary structure elements are displayed on the top. Average values and the respective standard deviations for the protease domain and the PDZ domain are indicated. **d**) $S^2_{axis}\bullet\tau_C$-values of peptide bound HtrA2$^{S306A}$ plotted against the HtrA2 amino acid sequence. Secondary structure elements are displayed on the top. Average values and the respective standard deviations for the protease domain and the PDZ domain are indicated. For comparison, values for corresponding residues in the apo state and the average values are indicated in faint blue and grey, respectively. **e)** $R_{2eff}$ values for the methyl-groups of apo MALVI$^{proS}$-HtrA2$^{S306A}$ at 313 K at the indicated frequencies, obtained from a methyl MQ CPMG relaxation experiment run at 18.8 T (top). The secondary structure elements are indicated on top. $\Delta R_{2eff}$ rates of the methyl-groups of MALVI$^{proS}$-HtrA2$^{S306A}$ are displayed in the bottom panel and the average is indicated. **f**, **g**) $R_{2eff}$ values for the methyl-groups of apo MALVI$^{proS}$-HtrA2$^{S306A}$ at 313 K (**f**) and $R_{2eff}$ values for the methyl-groups of peptide-bound MALVI$^{proS}$-HtrA2$^{S306A}$ at 313 K (**g**) for direct comparison of the $R_{2eff}$ values which could be determined in both states. Dashed lines represent average values at 750 Hz. **h**) Difference of the methyl group $\Delta R_{2eff}$ rates of the apo and the peptide bound holo state of MALVI$^{proS}$-HtrA2$^{S306A}$. Error bars indicate the standard fitting error obtained from the nonlinear least-squares minimization of one experiment ($n$ = 1) (panels **c–h**). **i**) Density of maximally frustrated

contacts in HtrA2 (PDB-ID: 5M3N) plotted along the HtrA2 residue number. The data was obtained with the Frustratometer Web Server [11]. Secondary structure elements are denoted by the grey shades. **j**) Clusters of maximally (red) and minimally (green) frustrated contacts denoted by the dashed lines on the structure of the HtrA2 protomer (PDB-ID: 5M3N) based on the data of the local structural frustration analysis shown in panel **j**. Source data are provided as a Source Data File.

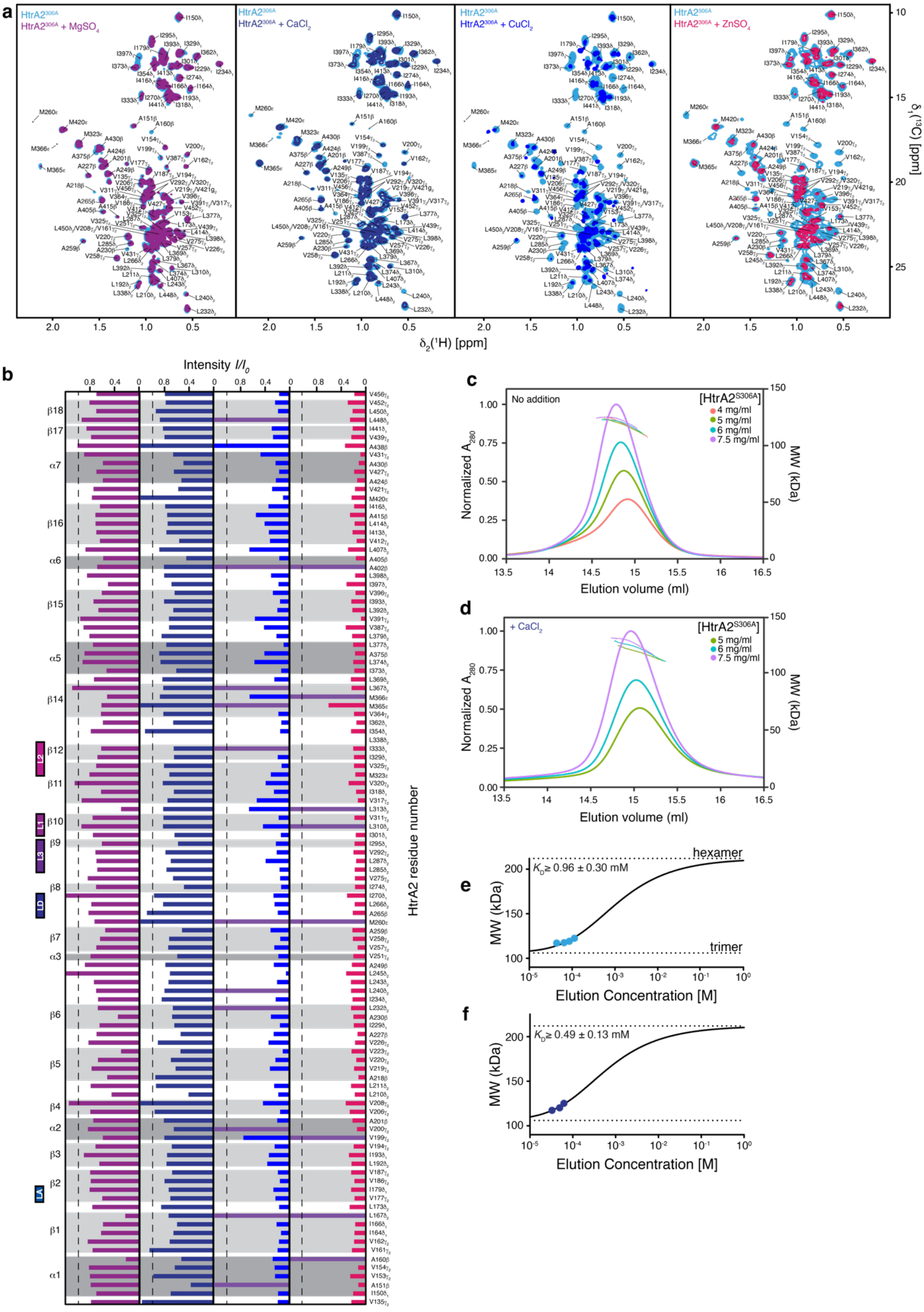

**Supplementary Fig. 18. Effects of divalent cations on HtrA2$^{S306A}$.**

**a)** [$^{13}C$,$^{1}H$]-NMR spectra of 100 µM HtrA2$^{S306A}$ MALVI$^{proS}$ (light blue) after addition of 25 mM $MgSO_4$ (purple), 25 mM $CaCl_2$ (dark blue), 2 mM $CuCl_2$ (blue) and 2 mM $ZnSO_4$ (magenta). **b)** Signal attenuations upon interaction with the different divalent cations as indicated in panel **a**. **c, d**) SEC elution profiles plotted as normalized absorbance ($A_{280}$) (solid lines, left axis) and MALS apparent molecular mass (dotted lines, right axis) as a function of the indicated protein concentrations without additive (**c**) or with $CaCl_2$ (**d**). **e, f)** Nonlinear regression fit of the averaged molar mass as a function of the elution concentration to a monomer-dimer equilibrium for HtrA2$^{S306A}$ in buffer without added metal ions (**e**) and in buffer with 25 mM $CaCl_2$ (**f**). Dotted lines represent trimer and hexamer molecular masses, respectively. Estimated molecular weights are reported in **Supplementary Table 5.** Source data are provided as a Source Data File.

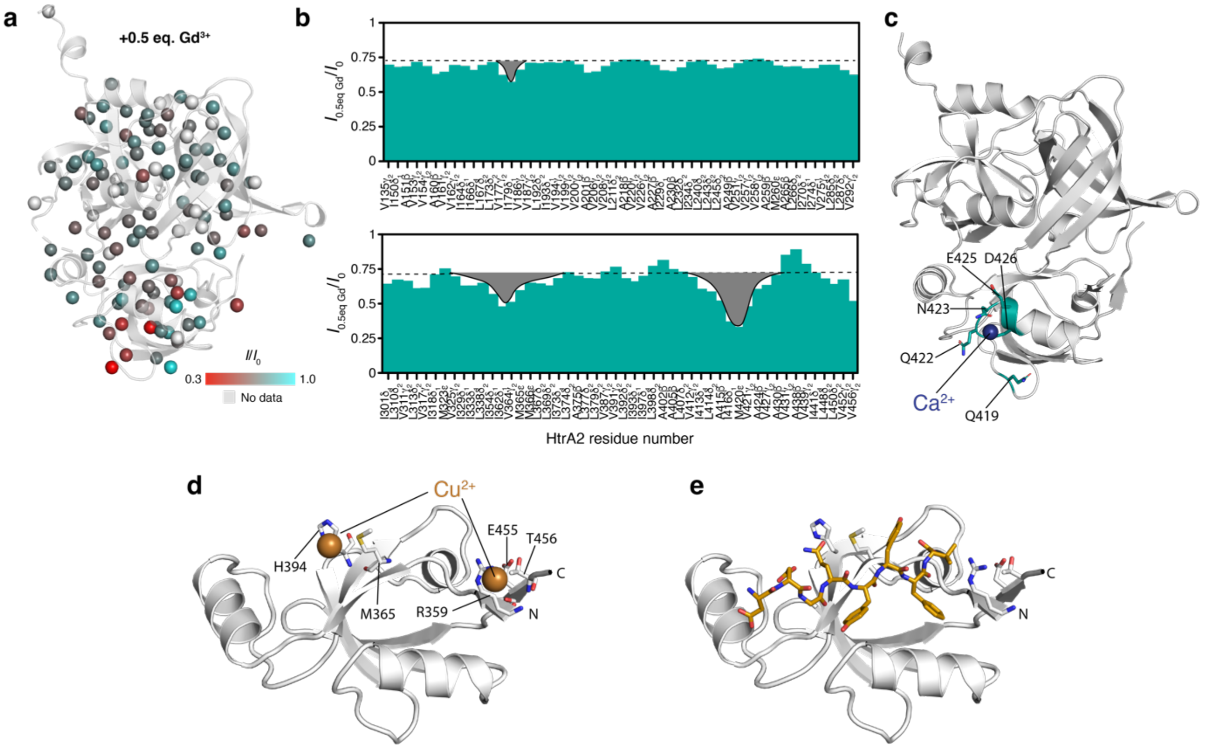


**Supplementary Fig. 19. $Gd^{3+}$ titration reveals a $Ca^{2+}$ binding site within the PDZ domain**.

**a**) Line broadening in response to titration of 0.5 molar equivalents of $Gd^{3+}$ towards full-length HtrA2$^{S306A}$ (PDB-ID: 5M3N). **b**) Signal attenuations following titration of $Gd^{3+}$ towards full-length HtrA2$^{S306A}$ plotted against the HtrA2 residue number. The grey shaded regions indicate affected regions. Errors were estimated based on the spectral noise of one measurement ($n$ = 1). Source data are provided as a Source Data File. **c)** Putative binding site of $Ca^{2+}$ to the HtrA2-PDZ domain based on our experimental

data and prediction of $Ca^{2+}$ binding sites in wild-type HtrA2 (PDB-ID: 5M3N) using MIB2 [12]. **d)** Putative binding sites of $Cu^{2+}$ to the HtrA2-PDZ domain based on the prediction of $Cu^{2+}$ binding sites in wild-type HtrA2 (PDB-ID: 5M3N) using MIB2 [12]. **e**) Structural model of the HtrA2-PDZ domain and the DD-PDZOpt peptide (yellow) complex in comparison to the residues involved in $Cu2^{+}$ binding as identified *in silico*. This panel represent the HADDOCK model shown in **Supplementary Fig. 12h**.

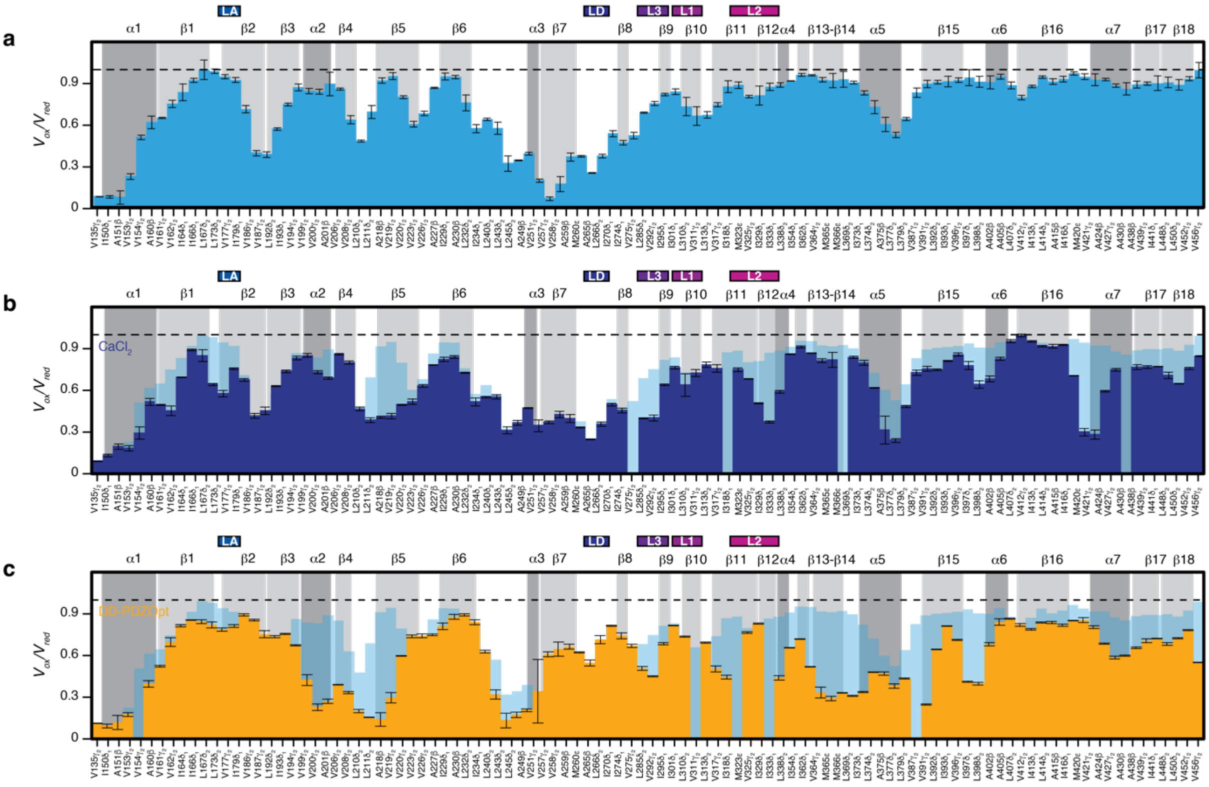


**Supplementary Fig. 20. Paramagnetic relaxation enhancement (PRE) of a spin label attached to HtrA2 at position S145C.**

**a–c**) PRE data for HtrA2$^{S306A, S145C}$ in buffer (blue) compared to HtrA2$^{S306A, S145C}$ in the presence of $CaCl_2$ (dark blue) and HtrA2$^{S306A, S145C}$ in the presence of DD-PDZOpt (yellow). The PRE is shown as the ratio $V_{ox}/V_{red}$, where $V_{ox}$ is the cross-peak intensity before reduction of the spin label and $V_{red}$ is the peak intensity after reduction of the spin label. Secondary structural elements and regulatory loops are indicated. The dashed line indicates where the $V_{ox}/V_{red}$ ratio equals 1. Errors were estimated based on the spectral noise of one measurement ($n$ = 1). Secondary structure elements and regulatory loops are indicated on top of each panel. For reference the apo data is additionally shown in faint blue also in panels **b** and **c**. Source data are provided as a Source Data File.

**Supplementry Tables**

**Supplementary Table 1.** Calculated molecular weights as well as estimated errors (S.D.) determined by SEC-MALS for HtrA2-PDZ.

| | **No additive** | **$CaCl_2$** | **$MgSO_4$** | **$CuCl_2$** | **$ZnSO_4$** | **DD-PDZOpt** |
|---|---|---|---|---|---|---|
| **[PDZ] μM** | **MW (kDa)** | **MW (kDa)** | **MW (kDa)** | **MW (kDa)** | **MW (kDa)** | **MW (kDa)** |
| **100** | 11.7 ± 0.74 | 11.7 ± 0.20 | 12.0 ± 0.17 | 11.9 ± 0.26 | 14.1 ± 0.44 | n.d |
| **150** | 11.7 ± 0.69 | 11.8 ± 0.18 | 11.8 ± 0.28 | 12.2 ± 0.34 | 14.3 ± 0.24 | 11.8 ± 0.06 |
| **250** | 11.8 ± 0.15 | 11.9 ± 0.06 | 12.2 ± 0.15 | 12.2 ±0.29 | 15.2 ± 0.24 | n.d |
| **350** | n.d | n.d | n.d | n.d | n.d | 12.0 ± 0.07 |
| **500** | 12.1 ± 0.15 | 12.3 ± 0.07 | 12.7 ± 0.18 | 12.7 ± 0.13 | 17.0 ± 0.30 | 12.1 ± 0.06 |
| **800** | 12.4 ± 0.05 | 12.7 ± 0.05 | 13.3 ± 0.15 | 13.3 ± 0.19 | 18.2 ± 0.35 | n.d |
| **900** | 12.6 ± 0.05 | n.d | n.d | n.d | n.d | n.d |

**Supplementary Table 2.** Input parameters for HADDOCK docking and resulting output parameters for the highest ranked structural cluster.

| **HADDOCK input parameters** | |
|---|---|
| Number of active residues[a] | |
| HtrA2-PDZ | 10 (I362, V364, L367, T368, V391, I393, I397, V412, I413, A424) |
| DD-PDZOpt | 8 |
| **HADDOCK output[b]** | |
| HADDOCK Score | - 93.1 ± 7.6 |
| Cluster Size | 67 |
| Average pairwise backbone RMSD within the cluster | 0.99 ± 0.2 Å |
| Buried Surface Area | 1116.7 ± 70.4 Å$^2$ |

[a] methyl bearing residues that showed significant chemical shift perturbations upon interaction on HtrA2-PDZ side as well as all DD-PDZOpt residues
[b] parameters determined for the ten lowest energy structures in the top cluster

**Supplementary Table 3.** Kinetic parameters determined for wild-type HtrA2 towards an optimized substrate peptide H2Opt in the absence or presence of divalent cations. Assay was performed using varying substrate concentrations, a fixed concentration of the activating peptide DD-PDZOpt at 50 μM and a fixed HtrA2 concentration of 510 nM (monomeric concentration). Kinetic parameters and respective errors (S.D.) were determined using InterferENZY [9].

| **Buffer type** | $K_M$ **(μM)** | $v_{max}$ **(M/s)** | $k_{cat}$ **($s^{-1}$)** | $k_{cat}/K_M$ **($M^{-1}$ $s^{-1}$)** |
|---|---|---|---|---|
| **No additive** | 8.06 ± 4.89 | $1.16 \cdot 10^{-10}$ ± $3.33 \cdot 10^{-11}$ | 0.00068 | 84.66 |
| **$CaCl_2$** | 4.2 ± 1.72 | $2.52 \cdot 10^{-10}$ ± $4.7 \cdot 10^{-11}$ | 0.00148 | 352.94 |
| **$MgSO_4$** | 2.74 ± 1 | $7.02 \cdot 10^{-11}$ ± $9.86 \cdot 10^{-12}$ | 0.00041 | 150.71 |
| **$CuCl_2$** | n.d | n.d | n.d | n.d |
| **$ZnSO_4$** | n.d | n.d | n.d | n.d |

**Supplementary Table 4.** Kinetic parameters determined for wild-type HtrA2 towards an optimized substrate peptide H2Opt in the absence or presence of divalent cations. Assay was performed using varying DD-PDZOpt concentrations, a fixed concentration of H2Opt at 20 μM and a fixed HtrA2 concentration of 510 nM (monomeric concentration). Kinetic parameters and respective errors (S.D.) were determined using GraphPad Prism 9.

| Buffer type | $K_{D,\mathrm{app}}$ (μM) | $v_{\mathrm{max}}$ (M/s) | $k_{\mathrm{cat}}$ ($s^{-1}$) | $k_{\mathrm{cat}}/K_{D,app}$ ($M^{-1}$ $s^{-1}$) |
|---|---|---|---|---|
| **No additive** | 27.9 ± 4.44 | $1.54 \cdot 10^{-9}$ ± $2.03 \cdot 10^{-10}$ | 0.0090 | 324.7 |
| **$CaCl_2$** | 1.53 ± 0.20 | $1.11 \cdot 10^{-9}$ ± $9.71 \cdot 10^{-11}$ | 0.0065 | 4276.9 |
| **$MgSO_4$** | 11.9 ± 3.65 | $3.36 \cdot 10^{-10}$ ± $5.46 \cdot 10^{-11}$ | 0.0019 | 166.1 |
| **$CuCl_2$** | n.d | n.d | n.d | n.d |
| **$ZnSO_4$** | n.d | n.d | n.d | n.d |

**Supplementary Table 5.** Calculated molecular weights as well as estimated errors (S.D.) determined by SEC-MALS for HtrA2$^{S306A}$.

| | **No addition** | **$CaCl_2$** |
|---|---|---|
| **HtrA2$^{S306A}$ (mg/ml)** | **MW (kDa)** | **MW (kDa)** |
| **7.5** | 122.5 ± 0.38 | 125.3 ± 0.38 |
| **6.0** | 119.2 ± 0.36 | 120.2 ± 0.36 |
| **5.0** | 117.6 ± 0.35 | 117.4 ± 0.35 |
| **4.0** | 117.2 ± 0.36 | n.d |

**Supplementary Table 6.** Plasmids used in this study.

| Plasmid | Tag |
|---|---|
| pET21b-HtrA2$^{S306A}$ | C-terminal 6xHis |
| pET21b-HtrA2 wild-type | C-terminal 6xHis |
| pET28b-HtrA2$^{349-458}$ (HtrA2-PDZ) | N-terminal 6xHis-SUMO |
| pET21b-HtrA2$^{S145C}$ | C-terminal 6xHis |

**Supplementary Table 7.** Primers used to generate the constructs used in this study.

| Plasmid | Primers |
|---|---|
| pET21b-HtrA2 wild type | 5'-TAACCAGGGGACCTCCACTGTTTCCAAAATCAATAGCTGCA-3'<br>5'-TGCAGCTATTGATTTTGGAAACAGTGGAGGTCCCCTGGTTA-3' |
| pET21b-HtrA2$^{S145C}$ | 5'-TGAAGTTGTACTGACACCGGGGAGAAGCGG-3'<br>5'-CCGCTTCTCCCCGGTGTCAGTACAACTTCA-3' |
| pET28b-HtrA2$^{349-458}$ | 5'-CGCATCTGGAACAGATTGGCGGTAATTCCTCCTCCGGAATCAGTG-3'<br>5'-CTTCACCTAAAGCATAAATTATTCTGTGACCTCAGGGGTCACATA-3' |

**Supplementary References**


1. Merski, M. *et al.* Molecular motion regulates the activity of the mitochondrial serine protease HtrA2. *Cell. Death Dis.* **8**, e3119 (2017).
2. Shen, Y. & Bax, A. Protein backbone and sidechain torsion angles predicted from NMR chemical shifts using artificial neural networks. *J. Biomol. NMR* **56**, 227–241 (2013).
3. Dosset, P., Hus, J. C., Blackledge, M. & Marion, D. Efficient analysis of macromolecular rotational diffusion from heteronuclear relaxation data. *J. Biomol. NMR* **16**, 23–28 (2000).
4. Lipari, G. & Szabo, A. Model-free approach to the interpretation of nuclear magnetic resonance relaxation in macromolecules. 1. Theory and range of validity. *J. Am. Chem. Soc.* **104**, 4546–4559 (1982).
5. Lipari, G. & Szabo, A. Model-free approach to the interpretation of nuclear magnetic resonance relaxation in macromolecules. 2. Analysis of experimental results. *J. Am. Chem. Soc.* **104**, 4559–4570 (1982).
6. Clore, G.M., Szabo, A., Bax, A., Kay, L.E., Driscoll, P.C., and Gronenborn, A.M. Deviations from the simple 2-parameter model-free approach to the interpretation of $^{15}$N nuclear magnetic-relaxation of proteins. *J. Am. Chem. Soc.* **112**, 4989–4991 (1990).
7. Waudby, C. A., Ramos, A., Cabrita, L. D. & Christodoulou, J. Two-dimensional NMR lineshape analysis. *Sci. Rep.* **6**, 24826 (2016).
8. de Vries, S. J., van Dijk, M. & Bonvin, A. M. J. J. The HADDOCK web server for data-driven biomolecular docking. *Nat. Protoc.* **5**, 883–897 (2010).
9. Pinto, M. F. *et al.* interferENZY: a web-based tool for enzymatic assay validation and standardized kinetic analysis. *J. Mol. Biol.* **433**, 166613 (2021).
10. Toyama, Y., Harkness, R. W., Lee, T. Y. T., Maynes, J. T. & Kay, L. E. Oligomeric assembly regulating mitochondrial HtrA2 function as examined by methyl-TROSY NMR. *Proc. Natl. Acad. Sci. USA* **118**, e2025022118 (2021).
11. Parra, R. G. *et al.* Protein Frustratometer 2: a tool to localize energetic frustration in protein molecules, now with electrostatics. *Nucleic Acids Res.* **44**, W356–W360 (2016).
12. Lu, C.-H. *et al.* MIB2: metal ion-binding site prediction and modeling server. *Bioinformatics* **38**, 4428–4429 (2022).
