# Supplementary material for: Structural basis of substrate recognition and allosteric activation of the proapoptotic mitochondrial HtrA2 protease
Source: Nat Commun. 2024 May 30;15:4592. doi: 10.1038/s41467-024-48997-5 (PMC11535027; doi:10.1038/s41467-024-48997-5)
Supplement: Supplementary file 3 — Reporting Summary [file 41467_2024_48997_MOESM3_ESM.pdf]

nature portfolio

Corresponding author(s): Björn M. Burmann

Last updated by author(s): Apr 18, 2024

Reporting Summary

Nature Portfolio wishes to improve the reproducibility of the work that we publish. This form provides structure for consistency and transparency in reporting. For further information on Nature Portfolio policies, see our Editorial Policies and the Editorial Policy Checklist.

Statistics

For all statistical analyses, confirm that the following items are present in the figure legend, table legend, main text, or Methods section.

n/a	Confirmed	
☐	☒	The exact sample size (n) for each experimental group/condition, given as a discrete number and unit of measurement
☐	☒	A statement on whether measurements were taken from distinct samples or whether the same sample was measured repeatedly
☒	☐	The statistical test(s) used AND whether they are one- or two-sided<br>*Only common tests should be described solely by name; describe more complex techniques in the Methods section.*
☒	☐	A description of all covariates tested
☒	☐	A description of any assumptions or corrections, such as tests of normality and adjustment for multiple comparisons
☐	☒	A full description of the statistical parameters including central tendency (e.g. means) or other basic estimates (e.g. regression coefficient) AND variation (e.g. standard deviation) or associated estimates of uncertainty (e.g. confidence intervals)
☒	☐	For null hypothesis testing, the test statistic (e.g. F, t, r) with confidence intervals, effect sizes, degrees of freedom and P value noted<br>*Give P values as exact values whenever suitable.*
☒	☐	For Bayesian analysis, information on the choice of priors and Markov chain Monte Carlo settings
☒	☐	For hierarchical and complex designs, identification of the appropriate level for tests and full reporting of outcomes
☒	☐	Estimates of effect sizes (e.g. Cohen's d, Pearson's r), indicating how they were calculated

Our web collection on statistics for biologists contains articles on many of the points above.

Software and code

Policy information about availability of computer code

Data collection	NMR data were collected on Bruker spectrometers operated with TOPSPIN 3.6. SEC-MALS runs were performed using the ASTRA 7.1.3 software (Wyatt Technology).
Data analysis	NMR data were processed using the mddNMR2.6 and NMRPipe software packages. Resonance assignments and spectral analyses were done in CARA. Secondary chemical shifts were calculated relative to the random coil values by the POTENCI algorithm as well as by using TALOS-N. Nonlinear least square fits of relaxation data were done with PINT 2.1. Analysis of the obtained relaxation rates was performed with Tensor2 on the NMRbox web server. Methyl-group relaxation dispersion data were fitted numerically to a two-site exchange model using the program ChemEx (available at https://github.com/gbouvignies/chemex/releases). NOE interaction patterns were generated and visualized using Flareplot (https://gpcrviz.github.io/flareplot/).For the docking of the activator peptide to the HtrA2-PDZ domain the HADDOCK web server was used. Molar mass, elution concentration, and mass distributions of SEC-MALS runs were calculated using the ASTRA 7.1.3 software (Wyatt Technology). SEC-MALS data was further analyzed using GraphPad Prism version 9.1.0. Quantification analysis of band intensities was made using the Image Lab software (Bio-Rad). Fluorescence cleavage assays were analyzed using either the webtool InterferENZY or GraphPad Prism version 9.1.0.

For manuscripts utilizing custom algorithms or software that are central to the research but not yet described in published literature, software must be made available to editors and reviewers. We strongly encourage code deposition in a community repository (e.g. GitHub). See the Nature Portfolio guidelines for submitting code & software for further information.

Data

Policy information about availability of data

All manuscripts must include a data availability statement. This statement should provide the following information, where applicable:

- Accession codes, unique identifiers, or web links for publicly available datasets
- A description of any restrictions on data availability
- For clinical datasets or third party data, please ensure that the statement adheres to our policy

All data needed to evaluate the conclusions in the paper are present in the manuscript and/or the Supplementary Materials. Uncropped gel images are provided in a Source Data file. The sequence-specific NMR resonance assignments for HtrA2-PDZ was deposited in the BioMagResBank (www.bmrb.wisc.edu) under accession code 51320. The NMR data used for the relaxation analysis, chemical shift perturbations, and NOE networks have been tabulated and are available alongside the fluorescence data on Mendeley data (http://dx.doi.org/10.17632/fgpcmp2kkg.1). Structure coordinates used in this study are available for the RCSB Protein Data Bank (https://www.rcsb.org) under accession codes 1JXQ, 1LCY, 1NW9, 2PZD, and 5M3N. Source data are provided with this paper.

Research involving human participants, their data, or biological material

Policy information about studies with human participants or human data. See also policy information about sex, gender (identity/presentation), and sexual orientation and race, ethnicity and racism.

Reporting on sex and gender	not applicable
Reporting on race, ethnicity, or other socially relevant groupings	not applicable
Population characteristics	not applicable
Recruitment	not applicable
Ethics oversight	not applicable

Note that full information on the approval of the study protocol must also be provided in the manuscript.

Field-specific reporting

Please select the one below that is the best fit for your research. If you are not sure, read the appropriate sections before making your selection.

[x] Life sciences [ ] Behavioural & social sciences [ ] Ecological, evolutionary & environmental sciences

For a reference copy of the document with all sections, see nature.com/documents/nr-reporting-summary-flat.pdf

Life sciences study design

All studies must disclose on these points even when the disclosure is negative.

Sample size	No statistical methods were used to predetermine sample size. Sample sizes were chosen in agreement with established procedures in the field.
Data exclusions	No data was excluded from the analyses.
Replication	Experiments were replicated (n > 2) to ensure reproducibility of the findings. The number of independent replicates for each experiment are specified in the respective Figure captions. All attempts at replication were successful. Assays were done at least in triplicates as stated within the MS.
Randomization	The experiments were not randomized, in agreement with established procedures in the field.
Blinding	The investigators were not blinded to allocation during experiments and outcome assessment, in agreement with established procedures in the field.

Reporting for specific materials, systems and methods

We require information from authors about some types of materials, experimental systems and methods used in many studies. Here, indicate whether each material, system or method listed is relevant to your study. If you are not sure if a list item applies to your research, read the appropriate section before selecting a response.

Materials & experimental systems

n/a	Involved in the study
☒	☐ Antibodies
☒	☐ Eukaryotic cell lines
☒	☐ Palaeontology and archaeology
☒	☐ Animals and other organisms
☒	☐ Clinical data
☒	☐ Dual use research of concern
☒	☐ Plants

Methods

n/a	Involved in the study
☒	☐ ChIP-seq
☒	☐ Flow cytometry
☒	☐ MRI-based neuroimaging

Plants

Seed stocks

Report on the source of all seed stocks or other plant material used. If applicable, state the seed stock centre and catalogue number. If plant specimens were collected from the field, describe the collection location, date and sampling procedures.

Novel plant genotypes

Describe the methods by which all novel plant genotypes were produced. This includes those generated by transgenic approaches, gene editing, chemical/radiation-based mutagenesis and hybridization. For transgenic lines, describe the transformation method, the number of independent lines analyzed and the generation upon which experiments were performed. For gene-edited lines, describe the editor used, the endogenous sequence targeted for editing, the targeting guide RNA sequence (if applicable) and how the editor was applied.

Authentication

Describe any authentication procedures for each seed stock used or novel genotype generated. Describe any experiments used to assess the effect of a mutation and, where applicable, how potential secondary effects (e.g. second site T-DNA insertions, mosiacism, off-target gene editing) were examined.
